# Temperate phages enhance bacterial host fitness via RNA-guided flagellar remodeling

**DOI:** 10.1038/s41564-026-02355-x

**Published:** 2026-05-27

**Authors:** Matt W.G. Walker, Egill Richard, Tanner Wiegand, Jing Wang, Zaofeng Yang, Americo A. Casas-Ciniglio, Florian T. Hoffmann, Hamna Shahnawaz, Ryan G. Gaudet, Nicholas Arpaia, Israel S. Fernández, Samuel H. Sternberg

**Affiliations:** 1 Department of Biological Sciences, Columbia University, New York, NY, USA.; 2 Department of Biochemistry and Molecular Biophysics, Columbia University, New York, NY, USA.; 3 Howard Hughes Medical Institute, Columbia University, New York, NY, USA.; 4 Simons Electron Microscopy Center, New York Structural Biology Center, New York, NY, USA.; 5 Department of Microbiology and Immunology, Columbia University, New York, NY, USA.; 6 Department of Genetics and Development, Columbia University, New York, NY, USA.; 7 Ikerbasque, Basque Foundation for Science, Bilbao, Spain.; 8 Instituto Biofisika (UPV/EHU, CSIC), University of the Basque Country, Leioa, Spain.; 9 These authors contributed equally.; 10 Present address: Can9 Bioengineering, New York, NY, USA.; 11 Present address: Division of Hematology, Department of Medicine, Stanford University, Stanford, CA, USA.

## Abstract

Bacterial flagella drive motility and play crucial roles in host-pathogen interactions, as flagellin is recognized by the mammalian immune system and flagellotropic bacteriophages. We recently discovered a family of phage-encoded, RNA-guided transcription factors called TldR that regulate flagellin expression, but the importance of this regulation to host fitness was unclear. Here we use a human clinical *Enterobacter* isolate encoding a Flagellin Remodeling prophage (FRφ) to show that FRφ exploits TldR and its flagellin isoform to alter the flagellar composition and phenotypic properties of its host. This transformation enhances bacterial motility and mammalian immune evasion, and cryo-EM structures reveal distinct flagellin architectures underlying physiological changes. FRφ also improves colonization in the murine gut, illustrating the beneficial effect of prophage-mediated flagellar remodeling in a host-associated environment. Collectively, our results reveal how RNA-guided transcription factors emerged in a parallel evolutionary path to CRISPR-Cas and were co-opted by phages to remodel the flagellar apparatus and enhance host fitness.

## INTRODUCTION

Tight and dynamic regulation of gene expression is fundamental to cellular and organismal function, occurring at both the transcriptional and post-transcriptional levels^1,2^. Transcription factors (TFs) typically regulate gene expression by binding specific genomic regulatory regions through protein-DNA interactions^3^, while post-transcriptional control often relies on small RNAs that recognize transcripts via RNA-RNA base pairing^4^. In contrast, we recently reported the widespread existence of programmable, RNA-guided TFs whose sequence specificity is conferred by RNA-DNA complementarity^5^. By exploring a family of transposon-encoded, RNA-guided nucleases within the TnpB superfamily — which are evolutionary precursors to CRISPR-Cas9 and Cas12^6,7^ — we uncovered multiple independent events of TnpB nuclease domain inactivation, without an apparent loss of guide RNA (gRNA) and DNA binding activities. Intriguingly, many of these TnpB-like nuclease-dead repressors (TldRs) evolved strong genetic associations with novel non-transposon genes, indicative of molecular domestication and functional coupling^5,8^.

**Fig. 1 |:**
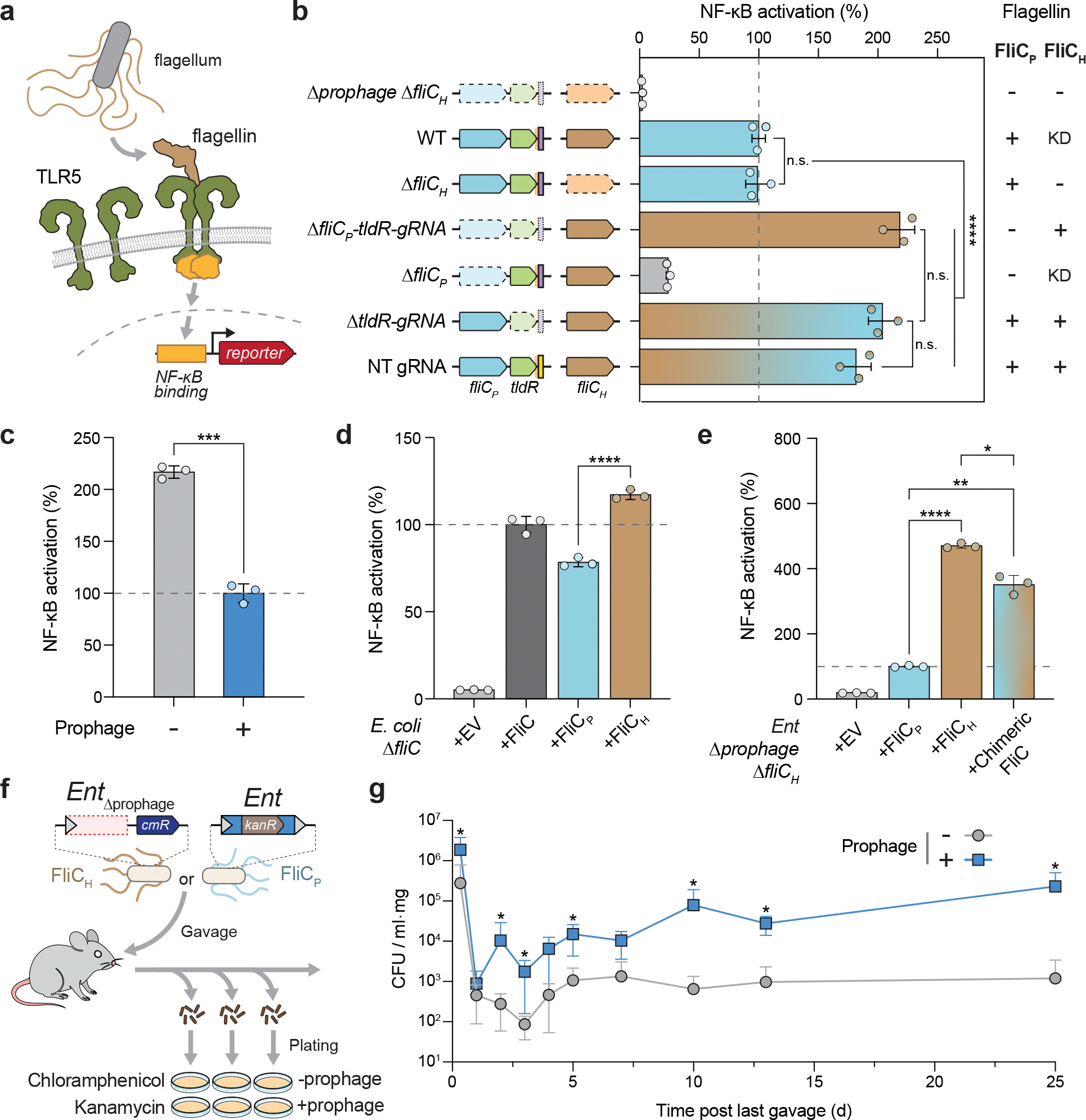
RNA-guided flagellin regulation in *Enterobacter* enhances cellular motility. **(a)** Schematic overview of TldR-mediated flagellar transformation. In the non-lysogenic state (top), the host-encoded flagellin gene *fliC*_*H*_ is actively transcribed and assembled into a native flagellum (brown). In the lysogen (bottom), a prophage-encoded RNA-guided transcription factor (TldR) represses *fliC*_*H*_ transcription, allowing expression of the prophage-encoded flagellin homolog *fliC*_*P*_, which assembles into a distinct flagellar filament (blue). This isoform switch has the potential to modulate bacterial motility, immune evasion, and susceptibility to secondary phage infection. **(b)** Representative soft agar plates imaged after 18 h of growth. The top image shows the wild-type lysogenic strain (WT), which exhibits a clear motility halo indicative of active flagellar function. The bottom panel shows a non-motile FliC– strain lacking both the host and prophage-encoded flagellin genes (Δ*fliC*_*H*_ *Δprophage*), resulting in complete loss of motility. **(c)** Bar graph quantifying bacterial motility via halo size measurements from experiments performed as in **B**, for the indicated strains. Bars indicate mean ± s.d. (n = 3 independent biological replicates, corresponding to independent bacterial cultures); **** p < 0.0001 (two-sided unpaired t-test). **(d)** Bar graph quantifying bacterial motility in *E. coli* AW405 or *Enterobacter* sp. BIDMC93 lacking endogenous flagellin, with episomal expression of either FliC_H_ or FliC_P_; an empty vector (EV) serves as a negative control. Bars indicate mean ± s.d. (n = 3 independent biological replicates, corresponding to independent bacterial cultures); * p < 0.05; *** p <0.001 (two-sided unpaired t-tests). **(e)** Bar graph quantifying bacterial motility in *E. coli* AW405 expressing TldR along with a guide RNA targeting the *fliC* promoter region. The schematic above illustrates the positions of specific mutations (black rectangles) relative to an invariant cognate target adjacent motif (TAM). Mutations within the seed region (nucleotides 1–6) de-repress FliC and thus lead to an increase in observed motility, indicating its necessity for efficient RNA-guided gene silencing. Bars indicate mean ± s.d. (n = 3 biological replicates, corresponding to independent bacterial cultures).

In one unusual TldR clade, we uncovered a novel flagellin (FliC) isoform adjacent to the TldR-gRNA cassette, and further identified both genes as residing within dormant, chromosomally integrated bacteriophages known as prophages^5^. Flagellin is the major extracellular structural component of the bacterial flagellum, a large membrane-embedded organelle that drives motility^9^. FliC is composed of a conserved D0/D1 core domain required for filament polymerization and motor function, flanked by highly variable, surface-exposed domains that diversify extensively across species^9^ . The TldR-associated flagellin homologs represent the first known example of prophage-encoded flagellin (hereafter FliC_P_), and are typically highly divergent from their host-encoded counterparts (FliC_H_) (Extended Data Fig. 1a). Remarkably, we revealed that in some clinical isolates of *Enterobacter*, prophage-encoded TldR specifically represses FliC_H_ expression by targeting its promoter^5^, allowing FliC_P_ to outcompete FliC_H_ and thus transform the flagellar identity of *Enterobacter* (Fig. 1a).

The integration of temperate phages into the bacterial chromosome — a process known as lysogenization — can lead to drastic host phenotypic changes, referred to as lysogenic conversion. In some cases, such as *Vibrio cholerae*^10^, lysogenic conversion has transformed non-pathogenic strains into potent human pathogens. Other prophage-conferred traits include enhanced biofilm formation^11^, improved cell invasion and intracellular survival^12^, phage resistance^13^, or competitive interactions with other bacteria^14^. However, rarely are lysogenic conversion events associated with alterations to host machinery as complex and fundamental as the flagellum, leading us to explore the range and nature of this remodeling event.

Here, we investigate how Flagellin Remodeling prophages (FRφ) impact host fitness by transforming their flagellar composition. We reveal that FRφ-driven remodeling enhances bacterial motility, decreases TLR5 recognition in human cells, and increases murine gut colonization, highlighting how phage-encoded organellar changes can profoundly influence host physiology. We further identify a distinct clade of TldR regulators in gut commensals linked to the translational regulator CsrA, suggesting a broader paradigm of coordinated transcriptional and post-transcriptional flagellin regulation.

## RESULTS

### RNA-guided flagellin regulation enhances motility

We set out to understand the biological consequences of RNA-guided flagellin regulation by examining bacterial motility on soft LB-agar plates, where motile strains form visible halos (Fig. 1b). We first compared *Enterobacter* sp. BIDMC93 (hereafter *Ent*) cells with and without the prophage encoding *fliC*_*P*_, using λ-Red-based recombineering to remove the 53.5-kb prophage and reconstitute the *attB* site (Methods). Lysogenic cells exhibited a striking motility enhancement (Fig. 1c). To identify the responsible prophage factors, we generated *fliC*_*P*_ or *tldR* knock-outs and reprogrammed the guide region to a non-targeting control (NT). Each mutation significantly reduced motility (Extended Data Fig. 1b) without affecting growth (Extended Data Fig. 1c), demonstrating that enhanced motility was dependent on prophage regulation of the host flagellin gene. RNA-seq confirmed strong expression of *tldR* and *fliC*_*P*_ in the native context (Extended Data Fig. 1d), and TldR ChIP-seq and RIP-seq experiments revealed native targeting to the *fliC*_*H*_ locus (Extended Data Fig. 1e) and association with its cognate gRNA (Extended Data Fig. 1f).

We next asked whether the increased motility was due to differences in flagellin expression or distinct biophysical properties of the filaments. Expressing either FliC_H_ or FliC_P_ from the same constitutive promoter in *E. coli* or *Enterobacter* cells lacking endogenous flagellin revealed a pronounced motility increase with FliC_P_ (Fig. 1d), indicating that the prophage-encoded flagellin filaments possess intrinsic motility-enhancing properties.

Next, we exploited the motility phenotype to define the molecular basis of TldR-mediated repression, hypothesizing that its activity would be governed by specific gRNA features as in other CRISPR systems^6,15^. Perturbing RNA-DNA complementarity revealed that guides as short as 6 nucleotides (nt) were sufficient to reduce motility (Fig. 1e), reminiscent of the Cas9/Cas12 seed sequence^16,17^, and systematic 2-nt mismatches further defined a 6-nt seed sequence (Extended Data Fig. 2a). Importantly, repression was also retained when testing naturally occurring guide-target mismatches from TldR homologs (Extended Data Fig. 2b).

### Architectural determinants of FliC_P_-dependent motility enhancement

**Fig. 2 |:**
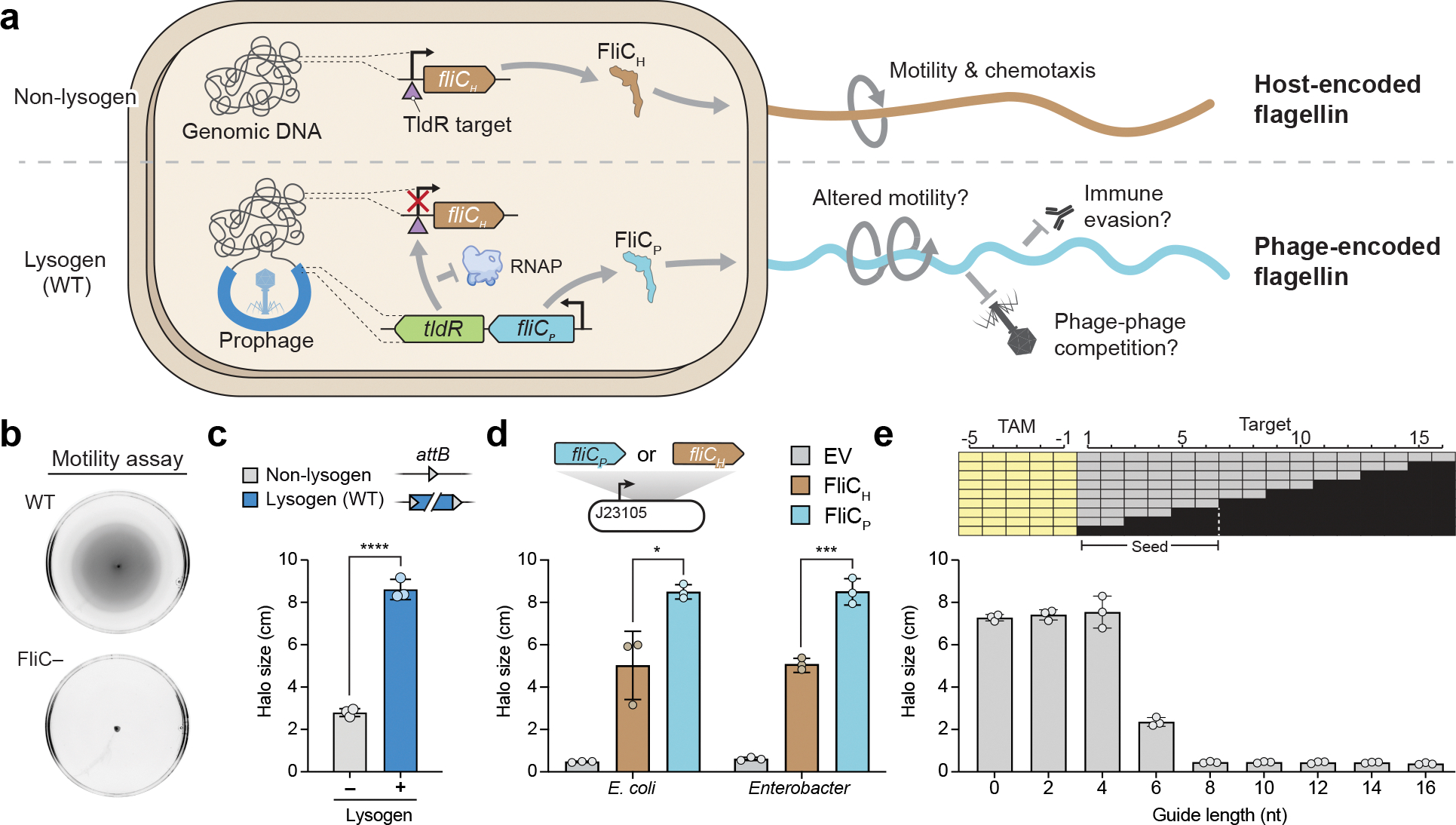
Cryo-EM structures of *Enterobacter* host- and prophage-encoded flagellin filaments. **(a**) SDS-PAGE analysis of the flagellar fraction from deflagellated *E. coli* cells expressing recombinant *Ent*FliC_H_ (top) or *Ent*FliC_P_ (bottom), shown alongside a protein molecular weight ladder. Image shown is representative of two independent repeats. **(b)** Domain annotations and cryo-EM structures of *Ent*FliC_H_ (top) and *Ent*FliC_P_ (bottom) monomers. Constant (D0–D1) and hypervariable (D2–D4) domains are labeled. **(c)** Molecular surface representation of *Ent*FliC_H_ (left) and *Ent*FliC_P_ (right) filaments from cryo-EM data, shown in both cross-sectional (top) and side (bottom) views. **(d)** Inter-subunit contact heatmaps for *Ent*FliC_H_ (top) and *Ent*FliC_P_ (bottom) filaments. Each heatmap quantifies the number of atomic contacts between thirty monomers within the respective filament, defined as any pair of atoms with distance ≤ 5 Å. This cutoff was selected to capture a broad range of non-covalent interactions, including van der Waals contacts, aromatic stacking, and proximal side chain interactions. (**e**) Cryo-EM structural model of two interacting FliC_P_ monomers (light and dark blue), highlighting the interaction region (inset, orange) and flexible loop (red) used for the experiments in panel (F). (**f**) Bar graph quantifying bacterial motility in an *Enterobacter* strain lacking endogenous flagellin, with episomal expression of FliC_H_, FliC_P_, or FliC_P_ variants harboring the indicated mutations. The empty vector (EV) serves as a negative control, and the chimeric construct substitutes the variable domain of FliC_H_ with that of FliC_P_. Bars indicate mean ± s.d. (n = 3 biological replicates).

We leveraged cryo-EM to visualize differences between FliC_P_ and FliC_H_ filaments that might explain their distinct motility phenotypes. We purified flagellin filaments by expressing both flagellin isoforms in an *E. coli ΔfliC* strain, mechanically shearing filaments from cells, and isolating filaments via centrifugation (Methods) (Fig. 2a). Both filament structures were determined using helical reconstruction in Relion5^18^, with the host FliC_H_ filament reconstructed with helical symmetry to an overall resolution of 3.7 Å, and the prophage FliC_P_ filament reconstructed to 7 Å using a hybrid approach (Fig. 2b,c, Extended Data Fig. 3, Methods).

FliC_P_ and FliC_H_ share a conserved core composed of highly similar D0 and D1 domains, but their external domains (D2–D4) differ markedly (Fig. 2b). The N- and C-termini of each FliC_P_ and FliC_H_ monomer interact to form long α-helical bundles that contact neighboring protomers, assembling with a periodicity of 11 monomers per turn around a ~25 Å pore that runs through the ~120 Å-wide core, which is used to export additional FliC copies during flagellar polymerization (Fig. 2c). Extending beyond these core regions, which bear strong similarity to flagella from *Escherichia coli* and *Salmonella enterica*^19^, variable domains project outward and account for the majority of the solvent-exposed surface (Fig. 2c). In FliC_H_ filaments, the external D2 and D3 domains form only minimal contacts with neighboring FliC_H_ subunits (Fig. 2d), creating threaded channels that run clockwise down the length of the ~240 Å-wide filament (Fig. 2c). In contrast, the external variable domains of FliC_P_ (D2, D3, and D4) form loops that extend dramatically from the flagellar core, shielding the inner filament within a much larger (~380 Å) reticulum of interacting subunits. FliC_P_ protomers form only minimal interactions with neighboring subunits through these variable domains, instead engaging in symmetric interfaces with subunits located roughly two helical turns apart (Fig. 2d,e). These extended loops impart flexibility to the mesh-like network surrounding the FliC_P_ core, contrasting the more rigid contacts formed between variable domains of adjacent FliC_H_ monomers (Extended Data Fig. 3e). Overall, FliC_P_ variable domains form substantially more inter-subunit than FliC_H_ filaments (Fig. 2d), which may enhance FliC_P_ filament stability.

To test whether these outer domain interactions contribute to enhanced motility, we performed targeted genetic disruptions. Replacing the entire FliC_P_ variable domain with that of FliC_H_ produced a chimera that perfectly phenocopied FliC_H_ motility (Fig. 2f). Targeted substitutions within a key interacting region (N319-N323) (Fig. 2e) likewise caused a dramatic loss of cellular motility (Fig. 2f, variants 1–3), whereas mutations in a distal region had no effect (Fig. 2f, variants 4–6), supporting a model in which FliC_P_ interactions within the variable domain enhance motility.

### FRφ is an infectious temperate phage that produces *Siphoviridae*-like virions

**Fig. 3 |:**
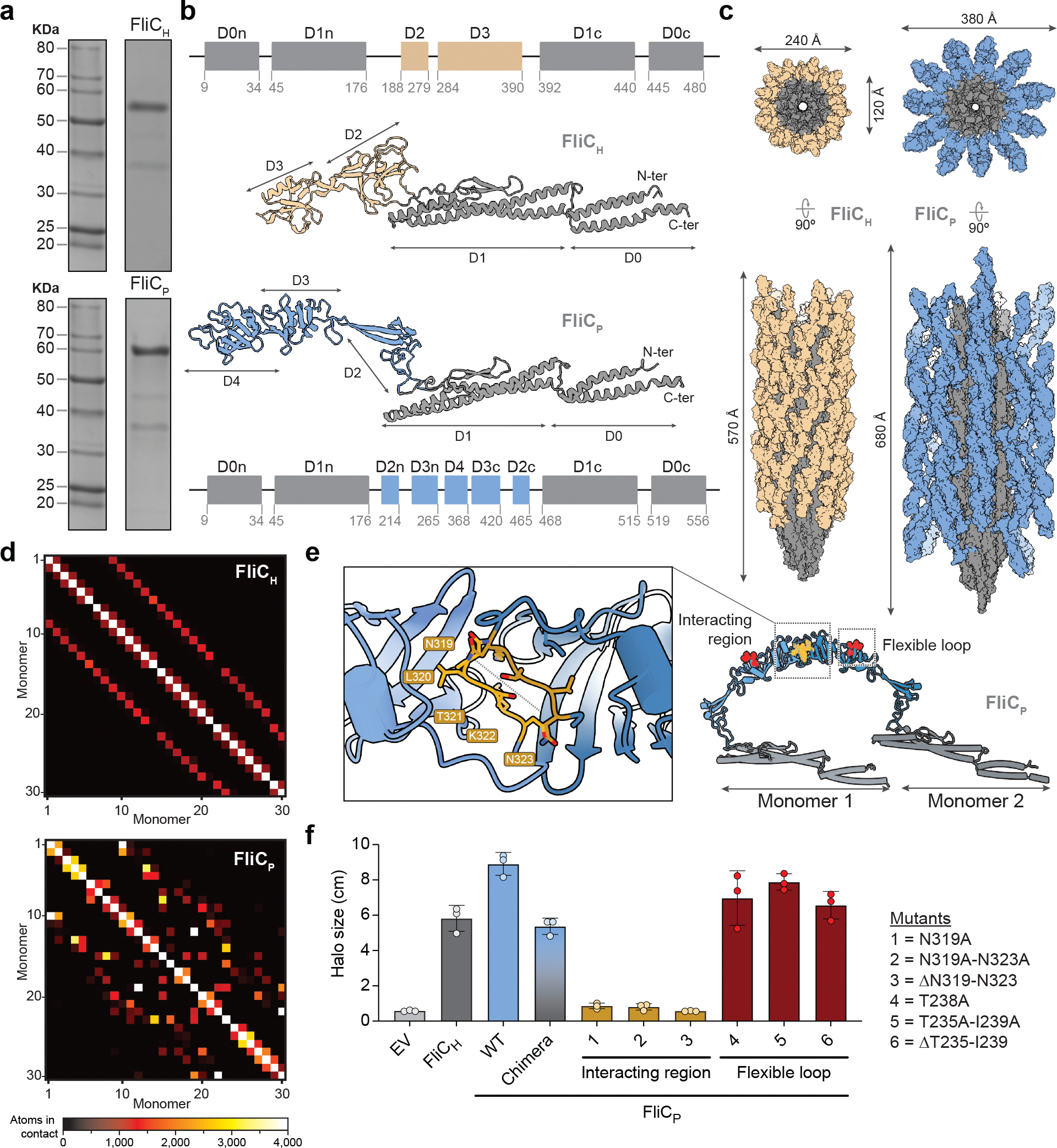
FRφ is a temperate, λ-like phage that modulates host flagellin composition. (**a**) Schematic of lysogenization assay, in which the prophage within the donor strain encodes a kanamycin resistance cassette (*kanR*, light green). The recipient strain lacks the prophage and encodes a chloramphenicol resistance cassette (*cmR*, blue) downstream of the attachment site (grey triangle), such that successful lysogenization events result in doubly-resistant clones. Prophage induction was induced with mitomycin C (MMC). **(b)** Representative plate images after the lysogenization assay in (a), demonstrating successful phage isolation and infection of naive recipient cells. Lysogenization efficiencies were indistinguishable when recipient cells lacked host flagellin (*ΔfliC*_*H*_). **(c)** Electron micrograph of FRφ. The scale bar represents 100 nm. **(d)** Representative microscopy images of cells before and after lysogenization, depicting the flagellar switch. Cells were co-labeled with FlAsH to detect TC-tagged FliC_H_, and streptavidin-Alexa Fluor 568 to detect AviTagged FliC_P_. The scale bar represents 5 μm.

Our prior genomics analysis^5^ suggested that the *tldR-fliC*_*P*_–containing prophage likely was active, so we next tested if it could enter the lytic phase and produce infectious viral particles. To detect lysogenization events, we employed a dual antibiotic resistance selection approach, in which we incorporated a kanamycin resistance cassette (*kanR*) into the prophage of a donor strain and a chloramphenicol resistance cassette (*cmR*) into the genome of a *Δprophage* recipient strain (Fig. 3a). We then treated donor cells with mitomycin C (MMC) to induce prophages, isolated phage particles by filtration, and mixed the filtrate with *Δprophage* recipient cells. After plating cells on double-antibiotic media, we detected CmR-KanR colonies, whereas no doubly resistant colonies were observed when the assay was performed with control cells lacking the prophage (Fig. 3b). We also observed lysogenization in the absence of MMC treatment to lesser extent (Extended Data Fig. 4a,b), indicating that the phage spontaneously produces infectious particles even under standard liquid growth conditions. To confirm the presence of integrated prophage genomes, we performed long-read whole genome sequencing and verified that the *kanR*-marked phage was integrated at the expected *attB* site (Extended Data Fig. 4c). To visualize the phage, we imaged the eluate by cryo-EM and observed phage particles with icosahedral heads and long, flexible, noncontractile tails — hallmarks of the *Siphoviridae* family^20^ (Fig. 3c). We thus designated this virus FRφ (Flagellin Remodeling phage), reflecting its unique regulatory function.

Importantly, our lysogenization assay also allowed us to investigate whether host flagella are required for infection. Many phages employ superinfection exclusion to prevent subsequent infection by the same or related phages^13^, and since some flagellotropic phages like χ use flagella as receptors^21^ , we hypothesized that TldR-mediated flagellar transformation might promote superinfection exclusion. However, infection of a non-flagellated recipient strain yielded similar lysogenization as a flagellated strain (Fig. 3b), ruling out flagellar transformation as a mechanism of superinfection exclusion.

Motivated by the hypothesis that FRφ infection triggers a physical remodeling of the flagellum, we next performed fluorescence microscopy using isoform-specific labeling to visualize the switch from FliC_H_ to FliC_P_. Established flagellin labeling strategies rely on cysteine-reactive maleimide dyes or the FlAsH system, which labels a genetically-encoded tetracysteine (TC) motif^22^. We developed an orthogonal *in vivo* biotinylation approach using the 15-amino acid AviTag, which can be endogenously biotinylated and detected with fluorescently labeled streptavidin^23^. We established this method in *E. coli*, scarlessly inserting the AviTag into *Eco*FliC at the site previously used for tetracysteine tagging. Incubation with Streptavidin-Alexa Fluor 568 yielded robust flagellar signal even without exogenous biotin or BirA (Extended Data Fig. 5a), and co-labeling with FlAsH confirmed the compatibility of both methods (Extended Data Fig. 5b), establishing the feasibility of multiplexed, isoform-specific flagellin labeling.

We then applied this approach in *Enterobacter* to visualize flagellar transformation during FRφ infection. We tagged *Enterobacter* FliC_H_ and FliC_P_ with the TC motif and AviTag, respectively, leveraging our cryo-EM structures to identify flexible regions and using a streamlined genome engineering method based on no-SCAR (Methods, Extended Data Fig. 5c,d). We then infected *Δprophage* recipient cells expressing TC-tagged FliC_H_ with phage particles isolated from donor cells expressing AviTagged FliC_P_, and imaged cells before and after infection. This dual-labeling strategy revealed a striking and complete switch in filament composition from FliC_H_ to FliC_P_ (Fig. 3d), providing direct visual evidence of flagellar transformation upon phage infection.

### FRφ encodes a tail fiber protein shufflon locus that exploits FhuA for cellular entry

**Fig. 4 |:**
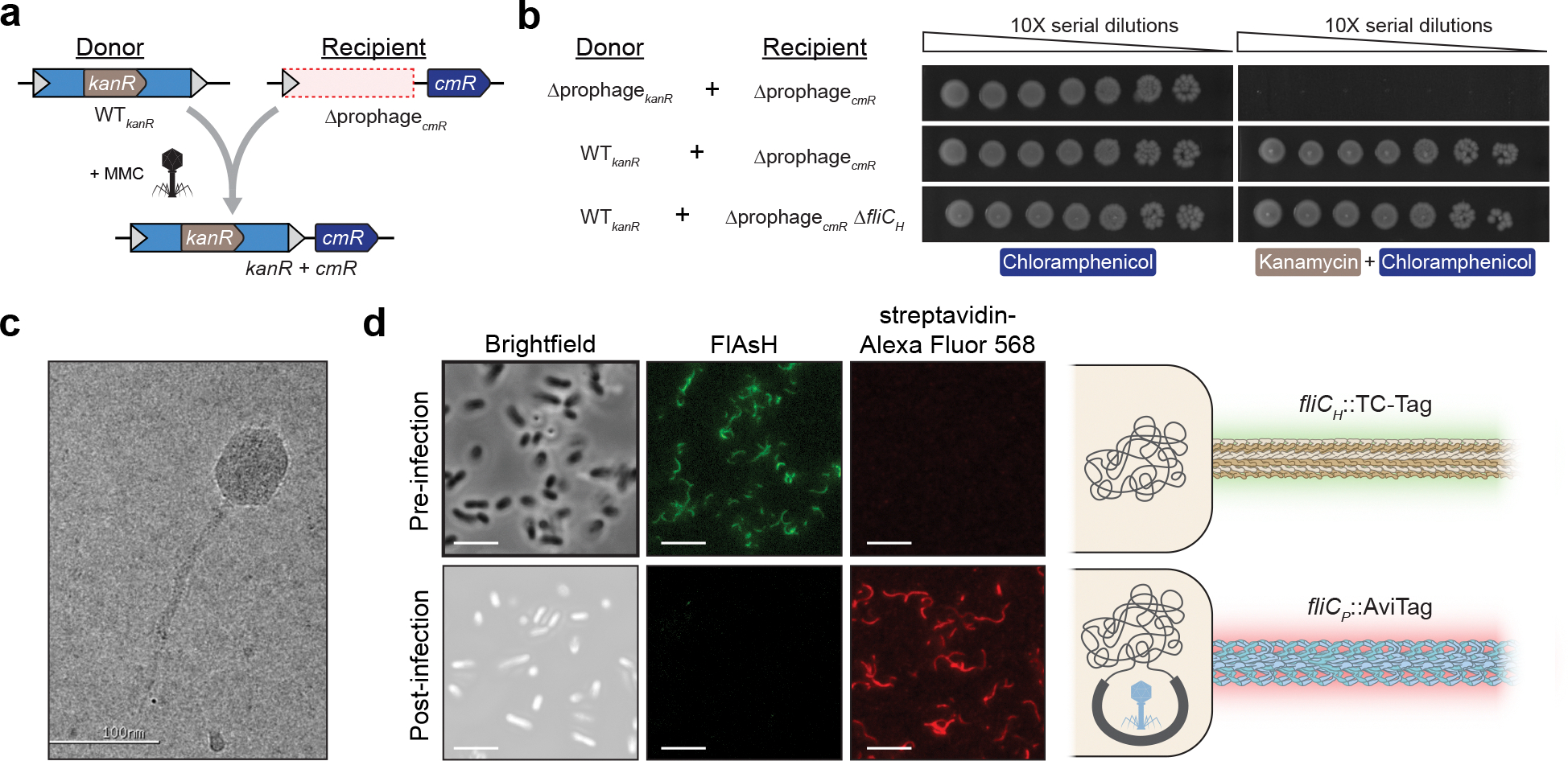
FRφ encodes a tail fiber protein shufflon locus that exploits FhuA for cellular entry. (**a**) Schematic of the FRφ-encoded shufflon tail fiber protein (TFP) locus, with AlphaFold 3 predictions of each TFP isoform. DNA segments capable of inversion are indicated at the top with circular arrows, and the distinct C-terminal domains are labeled C1–C3; *gin* encodes the responsible recombinase. **(b)** Quantification of the relative abundance of each TFP isoform from long-read sequencing of the WT FRφ prophage embedded in the *Ent* genome, isolated WT phage virions, and a prophage harboring a *Δgin* deletion mutation. Raw junction counts from HTS reads are listed in white text. **(c)** Representative plate images after lysogenization assay in a *Δgin* background, where TFP was locked into each of three distinct states harboring a unique C-terminal domain (C1, C2, or C3). Only phage particles expressing TFP-C2 can lysogenize *Ent*. **(d)** Comparison of phage λ lysogenic control locus and FRφ (top), and schematic depictions of *cI* / *cII* deletions to generate lytic/virulent FRφ (middle). Superpositions of homologous regions of CI, CII, and Cro are shown for phages λ and FRφ, depicted as AlphaFold 3 predictions, with RMSD values over *n* α-carbon atoms shown below each structural comparison; superpositions were calculated with the PDBeFold tool from EMBL-EBI. Regions covered by each superposition are indicated on the gene schematics above each predicted structure. **(e)** Plaquing assay of FRφ mutants *ΔcI* and *ΔcII* on a *Δprophage* strain of *Ent*, confirming that these mutations produce a lytic phage variant that generates clear zones of cell death. **(f)** Schematic illustrating the location and type of mutations in sequenced strains that are resistant to infection by FRφ. Three strains contain mutations in *fhuA*, while two contain mutations in *tonB*. **(g)** Plaquing assays demonstrate that FhuA complementation is necessary for FRφ infection in a *ΔfhuA* knockout strain, shown for both *Enterobacter* (left) and *E. coli* (right).

Long-read sequencing of our lysogenic *Ent* strain uncovered striking and unexpected heterogeneity within a predicted tail fiber protein (TFP) locus upstream of the *fliC*_*P*_-*tldR* region (Fig. 4a). Closer inspection revealed alternative 3′ coding sequences corresponding to three distinct C-terminal TFP isoforms (TFP-C1, -C2, and -C3), consistent with recombination events within a shufflon — a specialized locus known to undergo site-specific rearrangements that diversify the C-terminal domains of phage or pilus-associated proteins^24^ . Isoform quantification by sequencing confirmed that TFP-C1 was predominant (~50% of reads), while TFP-C2 and TFP-C3 each accounted for ~25% (Fig. 4b). This distribution was observed in both chromosomal and virion-derived DNA, and similar heterogeneity was detected in public sequencing datasets, highlighting limitations of genome assembly from non-clonal populations. The presence of a TFP shufflon in FRφ suggested a mechanism for tail fiber diversification, yet the host receptor(s) for these isoforms remained unknown. Having established that FRφ infection does not require flagella (Fig. 3b), we next sought to identify which TFP variant(s) mediate infection, and what cellular receptor(s) they target.

We returned to our lysogenization assay, but using a donor strain engineered to express a single, locked TFP variant, engineered by deleting the *gin* recombinase downstream of TFP; long-read sequencing of the *Δgin* donor strain confirmed the absence of TFP recombination (Fig. 4b). Intriguingly, phylogenetic analysis of the *gin* gene revealed its homology to the Tn3-family resolvase TnpR (Extended Data Fig. 6a, Table S1) and uncovered related loci encoding up to six TFP paralogs in other bacterial genomes (Extended Data Fig. 6b), underscoring the broader evolutionary complexity of TFP diversification.

With donor strains locked into each of the three possible TFP states (C1, C2, or C3), we purified isogenic phage populations and repeated our lysogenization assay. Strikingly, only the TFP-C2 phage was capable of forming doubly resistant colonies (Fig. 4c), identifying this variant as the sole isoform competent for *Ent* infection. This result not only revealed the functional specificity of TFP-C2 but also highlighted the potential for shufflon-mediated host-range modulation within the FRφ phage.

Next, to identify the host receptor used by FRφ, we generated a lytic version of the phage and screened for phage-insensitive *Enterobacter* mutants. We deleted the putative *cI* and *cII* repressor genes, identified by homology to the *λ* phage regulatory genes (Fig. 4d), confirmed the virulent (non-temperate) forms of FRφ by plaque assays (Fig. 4e), and isolated *Ent* colonies that were resistant to lytic infection. Long-read whole genome sequencing of five resistant clones revealed three putative loss-of-function mutations in *fhuA* and two in *tonB* (Fig. 4f). *fhuA* encodes a β-barrel outer membrane receptor previously implicated in phage entry for phages T1, T5, and phi80^25^, while *tonB* encodes a cytoplasmic membrane protein known to support the structural integrity and function of FhuA (Fig. 4f).

To validate that FhuA is required for FRφ infection, we engineered a Δ*fhuA* strain and observed a complete loss of plaque formation (Fig. 4g), whereas complementation with episomal *fhuA* restored infectivity (Fig. 4g). Remarkably, heterologous *fhuA* expression in *E. coli* MG1655 — which is normally resistant to FRφ — was also sufficient to license infection and lysis, demonstrating that FhuA alone is sufficient to confer susceptibility in a non-native host (Fig. 4g). However, attempts to establish lysogeny in *E. coli* expressing FhuA failed to yield lysogenized clones, implying that *Ent*-specific host factors govern the integration and/or maintenance of the prophage (Extended Data Fig. 4d,e).

Together, these findings identify FhuA as the key host receptor exploited by the TFP-C2 isoform of FRφ, linking shufflon-mediated tail fiber variation to host tropism.

### Screening for flagellotropic *Enterobacter* phage in environmental samples

Flagellotropic phages like χ use flagella as receptor^21^ , which represents a large (μm-size) extracellular appendage. Given this observation, and the dramatically different structures of FliC_H_ and FliC_P_, we hypothesized that the flagellar transformation orchestrated by TldR might act as a strategy to endow lysogenic bacteria with antiphage resistance against competing lytic, flagellotropic phages. However, no *Enterobacter*-specific phages with flagellotropy have, to our knowledge, been identified.

We employed two distinct high-throughput screening methods aiming at isolating novel *Ent*-specific flagellotropic phages from wastewater. The first method involved isolating plaques obtained after mixing wastewater samples with a *Δprophage* strain expressing host FliC_H_, and then identifying flagellin-specific phages as those that, after enrichment, were unable to infect isogenic strains *lacking* FliC_H_. However, all of these phages were similarly capable of infecting the *Δprophage ΔfliC*_*H*_ *Ent* strain (Extended Data Fig. 7a).

Our second method was adapted from Phage DisCo^26^ and involved co-infection of a mixed population of strains expressing FliC_H_/RFP or FliC_P_/GFP, thereby immediately revealing phages that selectively infect one strain over the other via the formation of green or red ‘plaques.’ We successfully adapted this method to screen flagellotropic phages and benchmarked it in *E. coli* (Extended Data fig. 7b) and in *Enterobacter* (Extended Data Fig. 7c). However, we again failed to isolate flagellotropic phages (Extended Data Fig. 7d). In total, we screened 1144 phages (632 by replica plaquing and 512 by Phage DisCo) but identified no flagellotropic *Enterobacter* phage, suggesting that such phages are either rare, absent from our sampled environments, or require alternative screening strategies for isolation.

### Flagellar remodeling promotes mammalian host immune evasion and improves gut engraftment

Having excluded superinfection exclusion and interphage competition as likely evolutionary forces behind flagellin remodeling, we next explored the impact on interactions with the mammalian innate immune system. Flagellin is detected as a key pathogen-associated molecular pattern (PAMP) by TLR5 extracellularly^27^ and NAIP5 intracellularly^28^, and some bacterial pathogens regulate flagellin to enhance colonization and pathogenesis. *Enterobacter* species from the *cloacae* complex, including *Enterobacter* sp. BIDMC93, are commensals in the human gastrointestinal tract but can become pathogenic in immunocompromised individuals. Since *Ent* is not an intracellular pathogen but would be surveilled by the immune system via TLR5, we asked whether FRφ lysogenization would alter TLR5 detection and downstream inflammatory signaling.

**Fig. 5 |:**
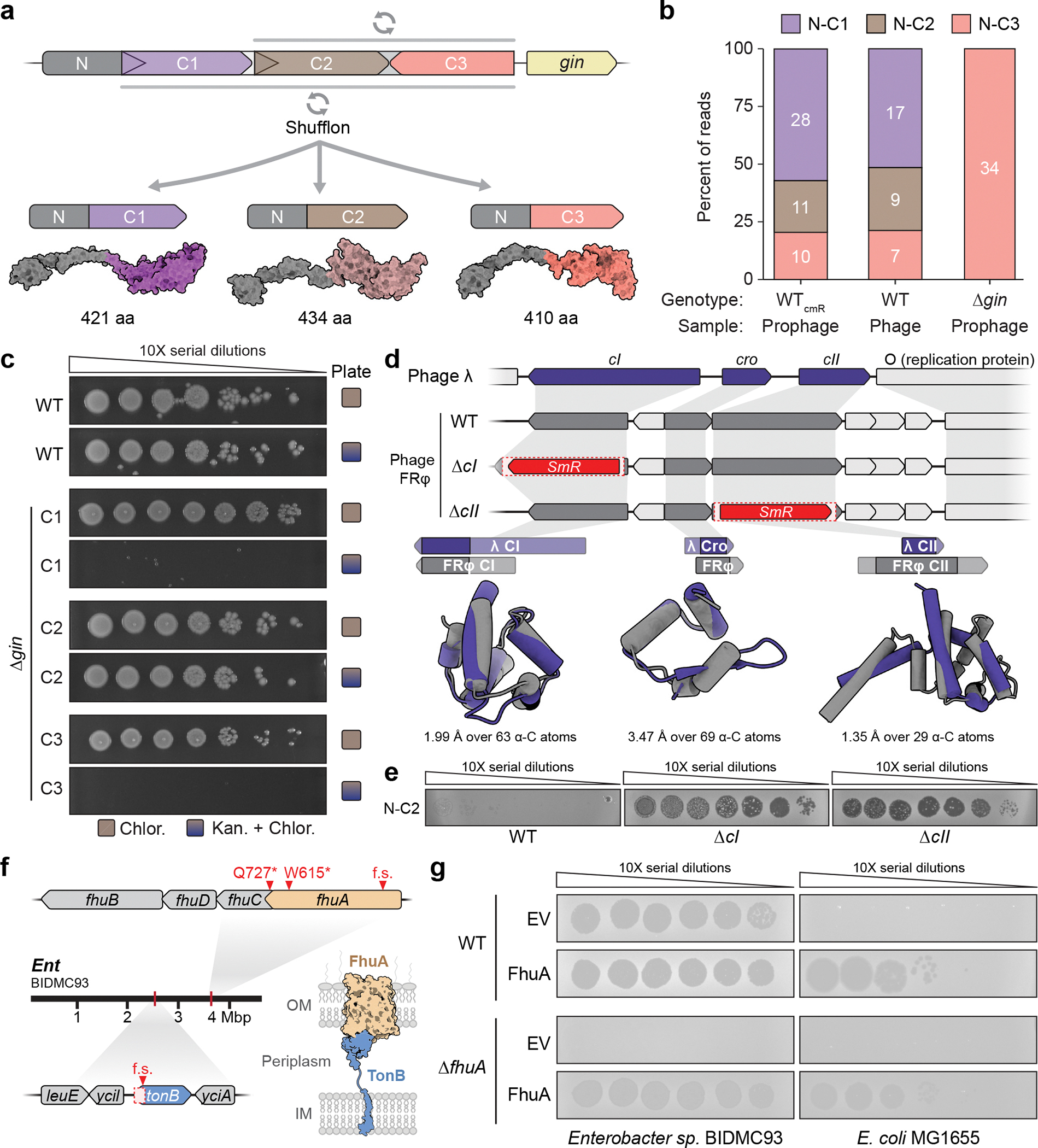
Phage-encoded flagellin attenuates activation of the mammalian host immune system and enhances engraftment. (**a**) Schematic of the TLR5 activation assay using TLR5 expressing HEK293T reporter cells. Readout: a colorimetric assay measuring NF-κB activation. **(b)** Panel of *Ent* mutants and TLR5-driven NF-κB activation in HEK293T cells, expressed as a percentage of the signal obtained with the WT lysogenic strain. The expressed flagellin isoform is indicated at right: absent (−), expressed (+), knocked-down by TldR (KD). Bars indicate mean ± s.d. (n = 3 biological replicates, corresponding to independent cell cultures). Statistical significance was assessed using one-way ANOVA with Dunnett’s multiple-comparisons tests (WT and *ΔfliC*_*H*_ as references, **** p-values < 0.0001). Selected pairwise comparisons were performed using two-sided unpaired t-tests; n.s., p-value > 0.05. **(c)** Bar graph quantifying TLR5 activation by *Ent* with and without the FRφ prophage. Bars indicate mean ± s.d. (n = 3 biological replicates, corresponding to independent cell cultures); *** p-value = 0.000131 (two-sided unpaired t-test) **(d)** Bar graph quantifying TLR5 activation by *E. coli* AW405 episomally expressing *E. coli* FliC, *Ent*FliC_H_ or *Ent*FliC_P_. Bars indicate mean ± s.d. (n = 3 biological replicates, corresponding to independent cell cultures); **** p-value = 5.648e-05 (two-sided unpaired t-test). **(e)** Bar graph quantifying TLR5 activation by *Ent* episomally expressing FliC_H_, FLiC_P_ or a chimeric flagellin consisting of the conserved FliC_P_ domains (D0-D1) and variable FliC_H_ domains (D2-D3). Bars indicate mean ± s.d. (n = 3 biological replicates, corresponding to independent cell cultures); **** p-value = 1.968e-05, ** p-value = 0.003951, * p-value = 0.01374 (two-sided unpaired t-tests). **(f)** Schematic of gut colonization assay, in which mice are gavaged with *Ent* strains containing or lacking the FRφ prophage. Each strain is marked with a antibiotic cassette, plating of bacteria from feces over time enables quantification of colonization. **(g)** Quantification of the mouse gut colonization assay, measured as colony-forming units (CFU) per mL⋅mg of resuspended feces for *Ent* strains with or without the prophage. Each point represents one mouse (n = 5 independent mice per group). Bars indicate mean ± s.d. Statistical significance was assessed using a two-sided Mann–Whitney U test with Benjamini–Hochberg false discovery rate (FDR) correction; *q < 0.05.

The epitope sequence that TLR5 binds^29^ is broadly conserved across FliC_P_ homologs within the conserved D1 domain (Extended Data Fig. 8a), but prior studies have demonstrated that this motif is not sufficient for TLR5 recognition and activation^30^. We therefore tested flagellin isoforms in a cellular model of TLR5 activation, using a human HEK293T cell line engineered to express TLR5 and a colorimetric reporter for NFκB-driven inflammation (Fig. 5a). When we stimulated TLR5-expressing HEK293T cells with the WT *Ent* lysogenic strain and compared it to a strain lacking any flagellin (*Δprophage* Δ*fliC*_*H*_), the WT triggered a robust TLR5-driven NF-κB activation signal, which we used as a baseline (Fig. 5b). Strikingly, a Δ*fliC*_*H*_ strain expressing only the prophage-encoded FliC_P_ isoform elicited a similar signal, whereas a strain expressing only FliC_H_ (Δ*fliC*_*P*_-*tldR-gRNA*) triggered nearly twice the level of NF-κB activation. These data indicate that FliC_H_​-containing flagella induce stronger innate immune signaling than FliC_P_​-containing flagella *in vitro*. To prove that this immune modulation is due to TldR-mediated silencing of *fliC*_*H*_, we examined NF-κB activation in strains expressing only TldR, with or without its guide RNA. Silencing of *fliC*_*H*_ via TldR markedly reduced immune activation, while de-repression — either through deletion of the gRNA or substitution with a non-targeting (NT) sequence — restored the high NF-κB activation observed with FliC_H_ alone (Fig. 5b). Together, these results suggest that FRφ lysogeny reshapes flagellin expression in a way that reduces flagellin-dependent immune signaling *in vitro*.

We next deleted the entire prophage and found that the non-lysogenic strain exhibited substantially higher levels of TLR5-driven NF-κB activation (Fig. 5c), consistent with our targeted genetic perturbations of *fliC*_*H*_ and *fliC*_*P*_. To further confirm that these differences could be specifically ascribed to flagellin, we expressed FliC_H_ or FliC_P_ under episomal plasmid conditions in *ΔfliC* strains and found that FliC_P_ consistently elicited a weaker NF-κB activation signal than FliC_H_ (Fig. 5d,e). Intriguingly, a chimeric FliC comprising the conserved domains (D0–1) of FliC_P_ and variable domain (D2–3) of FliC_H_ largely phenocopied FliC_H_ (Fig. 5e), suggesting that the solvent-exposed outer domain of the flagellar filament, rather than the TLR5 recognition motif itself, contributes to differences in TLR5 binding and/or signaling.

Finally, we investigated how flagellar transformation might impact gut colonization. Mice were gavaged with isogenic *Ent* strains lacking or harboring the FRφ prophage with a deleted integrase (Δ*int*) to prevent excision (Methods), and fecal colonization was quantified over 25 days by selective plating (Fig. 5f). Remarkably, the lysogen expressing FliC_P_ achieved significantly higher abundances than the non-lysogen expressing FliC_H_, at all time points after 24 hours (Fig. 5g). Control experiments confirmed the absence of pre-existing resistant strains (Extended Data Fig. 8b) and equivalent gavage efficiencies across groups (Extended Data Fig. 8c). Importantly, increased persistence of the lysogen was not associated with pathogenicity, as both groups gained weight normally (Extended Data Fig. 8d). Because flagella contribute to early biofilm formation, which is an important determinant of gut colonization and persistence^31^, we examined whether flagellin identity influences biofilm formation (Extended Data Fig. 9a, Methods). No differences in biofilm formation were observed among *Ent* mutants compared to a non-flagellated strain *(Δprophage ΔfliC*_*H*_), indicating that flagellar composition does not affect biofilm formation under the tested conditions (Extended Data Fig. 9b,c). Although heterologous expression of FliC_H_ *in E. coli* modestly reduced biofilm formation relative to FliC_P_​ (Extended Data Fig. 9d), no effect was observed in Ent upon overexpression of these isoforms (Extended Data Fig. 9e). Together, these results indicate that enhanced colonization of prophage-containing cells is unlikely to be explained by differences in biofilm formation.

Collectively, our results highlight multiple axes of host-pathogen interactions that are manipulated through flagellar transformation by phage FRφ, with the infected bacterial host itself becoming a more competitive constituent of the microbiome within its corresponding mammalian host. This bacterial fitness advantage likely served as one of the driving forces behind evolutionary emergence of the phage-encoded *tldR-fliC*_*P*_ pathway for RNA-guided flagellin control.

### Dual modes of flagellin regulation by CsrA-associated TldRs

**Fig. 6 |:**
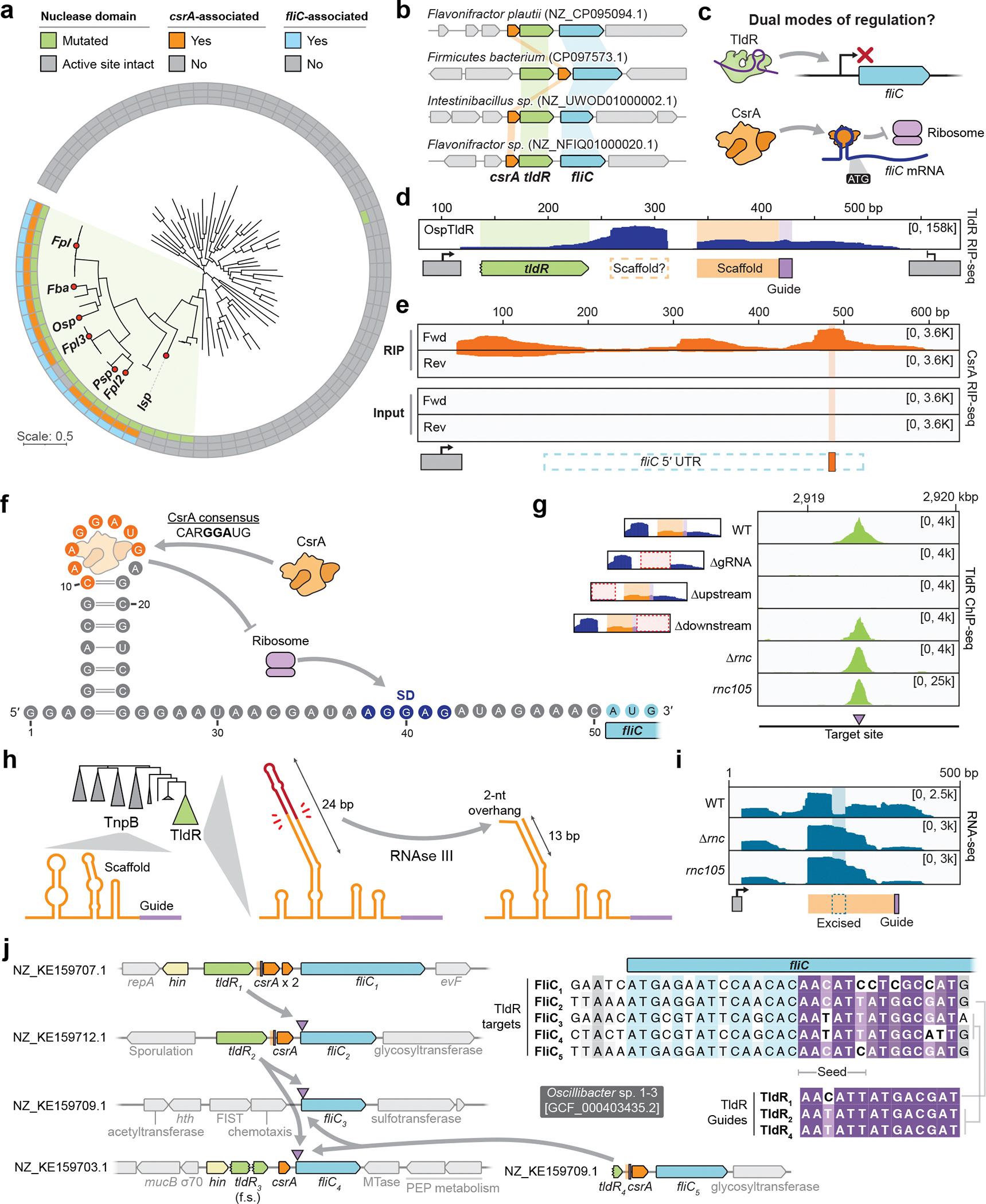
Dual modes of flagellin regulation by CsrA-associated TldRs **(a)** Phylogenetic tree of CsrA-associated TldRs. The inner ring highlights TldRs with mutations in the RuvC nuclease domain (green); the middle ring indicates *csrA* association (orange); the outer ring indicates *fliC* association (*hag* in *Firmicutes*). Red circles mark systems selected for functional testing. **(b)** Schematic of the genetic architecture of four *csrA*-associated *tldR* loci. **(c)** Model for dual flagellin regulation. TldR targets *fliC* to inhibit transcription, while CsrA targets the 5′ UTR of the *fliC* mRNA to inhibit translation. **(d)** RIP-seq coverage profiles of FLAG-tagged *Osp*TldR in *E. coli,* with the putative guide sequence highlighted (purple). Two enriched regions downstream of *tldR* correspond to the scaffold region of the guide RNA (orange). **(e)** RIP-seq coverage profiles of FLAG-tagged *Osp*CsrA in *E. coli*, mapped to the 5′ UTR of *fliC*. The orange highlight at bottom marks the predicted CsrA recognition motif (5′-CAAGGAUG-3′). **(f)** RNA secondary structure prediction of the *fliC* 5′ UTR, showing the CsrA motif (orange) within a stem-loop. The Shine-Dalgarno (SD) and start codon are highlighted in dark and light blue, respectively. **(g)** ChIP-seq coverage profiles of FLAG-tagged *Osp*TldR in *E. coli* at a genomic target site complementary to the guide sequence, shown across mutant backgrounds. Deletion of the guide RNA (ΔgRNA) or its upstream region (Δupstream) abolishes targeting, while deletion of the downstream region (Δdownstream) or RNase III inactivation (Δ*rnc*, *rnc105*) have no effect. **(h)** Predicted guide RNA structures from representative TnpB and TldR homologs, below a simplified phylogenetic tree from **(a)**. A schematic model of the TldR gRNA before and after RNase III cleavage shows activity facilitated by the expansion of a stem loop (red). **(i)** RIP-seq coverage profiles of FLAG-tagged *Osp*TldR in *E. coli* across the guide RNA, comparing WT, *Δrnc*, and *rnc105* strains. RNase III inactivation restores coverage in the dropout region, consistent with RNA processing. **(j)** Schematic of the genetic architecture of four distinct *tldR-csrA* loci present in *Oscillibacter*, suggesting multi-tiered *fliC* regulation. One locus contains frameshift mutations in the *tldR* gene (likely inactive), though it may still contribute gRNAs *in trans*. Putative TldR regulatory targets and gRNA guide sequences are shown.

While investigating FRφ, we identified a distinct clade of chromosomally-encoded TldRs genetically linked to both *fliC* and the post-transcriptional regulator *csrA*^5,8^ (Fig. 6a,b, Table S2), raising the possibility of a dual mode of flagellin regulation at the level of transcription and translation (Fig. 6c). Remarkably, RIP-seq and ChIP-seq analyses revealed that these host-encoded TldRs also employ gRNAs to target alternative flagellin genes, with target sites positioned directly within *fliC* open reading frames (Fig. 6d, Extended Data Fig. 10a–e, Table S3). Parallel CsrA RIP-seq datasets demonstrated specific enrichment at the 5′ UTR of the same *fliC* target gene putatively repressed by TldR (Fig. 6e), which contains a CsrA binding motif within a predicted stem-loop (Fig. 6f). When we ranked CsrA RIP-seq peaks, only two regions exhibited >3-fold enrichment relative to input — the fliC UTR and csrB (Extended Data Fig. 10f) — supporting the *fliC* UTR as a primary target.

In the course of analyzing RIP-seq data for an *Oscillibacter* TldR homolog, we unexpectedly observed two peaks in the gRNA region separated by a severe drop in read coverage (Fig. 6d), in contrast to the continuous coverage observed for other TnpB and TldR gRNAs^5,32^. Perturbation experiments revealed that the upstream sequence was essential for DNA target binding, whereas the downstream region was dispensable (Fig. 6g). The coverage gap corresponded to the distal end of a predicted stem-loop expected to generate a 2-nt 3′ overhang reminiscent of RNase III cleavage^33^ (Fig. 6h). RNA-seq in *rnc* mutant *E. coli* strains^34^ restored continuous gRNA coverage without disrupting DNA targeting (Fig. 6g,i), revealing an RNase III-dependent dual-guide RNA and suggesting a potential evolutionary intermediate of crRNA-tracrRNA systems (Type II and V CRISPR-Cas)^35,36^.

Intriguingly, genome analysis of a representative *Oscillibacter* strain revealed four *csrA-tldR* loci whose guide sequences predict extensive cross-targeting of *fliC* homologs (Fig. 6j), suggesting multiplexed regulation that coordinates transcriptional and translational control of multiple flagellin genes.

In a final conceptual parallel, we observed *hin* genes immediately upstream of most *csrA*-*tldR* loci. Hin invertases are homologous to Gin recombinases that swap bacteriophage tail fiber protein genes^37^ (Fig. 4a), leading us to search for local structural rearrangements. Comparative analysis of related loci from *Flavonifractor plautii* revealed ~120-bp DNA inversions immediately downstream of *hin*, in a region containing a FliA-dependent promoter (Extended Data Fig. 10g). In *Salmonella*, Hin-mediated recombination of a flagellin promoter inverton drives phase variation between alternative flagellin isoforms^38^. The conserved positioning of *hin* and DNA inversions upstream of *csrA–tldR* loci suggests an additional regulatory layer controlling flagellar isoform expression in TldR-encoding organisms (Extended Data Fig. 10h).

## DISCUSSION

TnpB proteins encoded within bacterial IS*200*/IS*605* and IS*607* elements represent a vast reservoir of RNA-guided nucleases^6–8^ that have been repeatedly domesticated over evolutionary timescales, giving rise to dozens of CRISPR–Cas12 subtypes involved in bacterial adaptive immunity^39^ and programmable transposition^40,41^. In parallel, mutational inactivation of the RuvC domain resulted in the evolution of TnpB-like nuclease dead repressors (TldR) that function as RNA-guided transcription factors^5^, akin to engineered CRISPR interference (CRISPRi) technologies^42^. Here, we focused on Flagellin Remodelling prophages (FRφ) that encode both a FliC_H_-targeting TldR and an alternative flagellin isoform, FliC_P_, effectively rewiring the flagellar machinery of lysogenized hosts. Using an integrative experimental approach in *Enterobacter*, we demonstrate that FRφ-driven flagellar reconfiguration enhances motility and reduces innate immune activation *in vitro*, correlating with increased colonization of the murine gut. This discovery highlights a major physiological shift driven by lysogenic conversion, with implications for both bacterial and mammalian hosts.

We initially envisioned that FRφ-mediated flagellar reprogramming might promote superinfection exclusion, as described for other phages^43,44^. One could hypothesize FRφ might bind FliC_H_-derived flagella as a receptor, then mask it after lysogeny by switching to FliC_P_. However, we found that FRφ, like T5 and ɸ80, instead recognizes FhuA. Intriguingly, the combinatorial tail fiber protein isoforms catalyzed by the shufflon cassette likely increase the FRφ host range by allowing recognition of multiple receptors. Despite screening >1,100 environmental isolates, we also failed to identify flagellotropic phages targeting *Ent*. Together, we conclude that flagellar transformation in *Ent* is unlikely to have evolved primarily for phage resistance.

We next sought to understand other selective advantages of FRφ-mediated lysogeny. Strikingly, we found that *Enterobacter* strains featuring prophage-derived flagella exhibited markedly enhanced motility. This effect was recapitulated when FliC_P_ was heterologously expressed in *E. coli*, indicating that the phenotype is intrinsic to the flagellin isoform. Structural analysis revealed that FliC_P_ assembles into a uniquely thick flagellar filament characterized by enlarged outer domains that wrap the filament core in a mesh-like configuration. Enhanced inter-protofilament contacts appear central to the motility phenotype, since mutagenesis at these interfaces diminished motility.

In addition to promoting motility, FRφ lysogeny is associated with reduced NF-κB activation in TLR5-expressing cells *in vitro*. Although the TLR5 epitope lies within the conserved D1 domain, FliC_H_ elicited approximately twice the level of TLR5 activation as FliC_P_, a difference preserved during heterologous expression in *E. coli.* Chimeric constructs in which the outer domains of FliC_P_ were replaced by those of FliC_H_ restored high TLR5 activation, suggesting that filament architecture, rather than primary sequence variation at the TLR5-binding site, contributes to differences in immune activation. We hypothesize that increased filament thickness and inter-protofilament interactions may limit flagellin shedding, reducing monomer availability for immune detection. This interpretation aligns with theoretical models of bacterial motility, which predict that filament thickness and stiffness are critical determinants of propulsion dynamics^45^, and with the observation that some bacteria exploit specific proteases to degrade extracellular FliC monomers, thereby evading immune detection^46^. In the case of FRφ, however, the strategy would be structural rather than enzymatic, with filament stabilization indirectly reducing monomer availability and modulating host immune activation.

FRφ lysogeny produces a consequential ecological outcome: enhanced colonization of the murine gut by *Enterobacter*. Given that both motility and host sensing are critical for host colonization^47,48^, FliC_P_-associated traits may contribute to this advantage, although disentangling their relative contributions remains a challenge. We also identified related RNA-guided flagellar regulatory modules in other gut commensals, where TldR-FliC loci associate with CsrA, suggesting dual transcriptional and post-transcriptional regulation. This regulation parallels established phase variation systems in gut-associated bacteria^49^ and may facilitate adaptation to heterogeneous intestinal environments^50^.

While TldR-FliC_P_ expression appears constitutive under the laboratory conditions tested in our study, we cannot exclude the possibility that expression varies *in vivo*. If so, FRφ lysogeny may provide not only structural flagellar remodeling, but also a mechanism for phase variation, enabling dynamic adaptation of motility and immune evasion in a tissue-specific and/or temporally controlled manner. Such a model would position FRφ as a modulator of host–microbe interactions, optimizing the fitness of its lysogenic host in fluctuating environments.

## METHODS

### Strain and plasmid construction.

Strains used in this study are listed Table S4. Genomic mutants of *E. coli* and *Enterobacter* were generated either by λ-Red recombineering or by an adapted protocol for scarless genomic edits using counterselection by *Sp*Cas9 (no-SCAR^51^). For strains constructed using λ-Red recombineering, mutants were designed to replace each gene of interest with an antibiotic resistance cassette (either kanamycin, chloramphenicol, or spectinomycin), which was amplified by PCR with Q5 High-Fidelity DNA Polymerase (NEB) using primers that contained at least 50-bp homology arms flanking the disrupted locus. PCR amplicons were resolved on a 1% agarose gel and purified (QIAGEN). Electrocompetent cells were prepared containing a temperature-sensitive plasmid that encodes the λ-Red machinery under the control of a temperature-sensitive promoter (pSL2684). Protein expression from the temperature-sensitive promoter was induced by incubating cells at 42 °C for 25 min immediately prior to electrocompetent cell preparation. Then cells were transformed with 200–600 ng of each insert (2 kV, 200 Ω, 25 μF) and recovered for at least 4 h in 3 ml of LB media. After recovery, cells were spread onto 100 mm standard plates with antibiotic (either 50 μg/ml kanamycin, 25 μg/ml chloramphenicol, or 200 μg/ml spectinomycin) and grown at 37 °C. Antibiotic-resistant colonies were genotypes by Sanger sequencing (GENEWIZ) to confirm each desired disruption.

For *E. coli* and *Enterobacter* strains constructed using *Sp*Cas9, we followed the protocol for Scarless Cas9 Assisted Recombineering (no-SCAR^51^), with some modifications that we found to be necessary for constructing genomic mutants of *E. coli* strain AW405. Spacers targeting the disrupted loci of interest were selected and cloned into a gRNA expression plasmid (pSL6050). Cells for the strain of interest were transformed with the gRNA expression plasmid at 30 °C (since it is temperature sensitive), then grown up to mid-log phase and induced with L-arabinose to 0.2% for 15 min. Cells were then made electrocompetent by washing twice in 10% glycerol, and co-transformed with ssDNA donor and ~250 ng Cas9 plasmid (pSL0411). The ssDNA donor was designed using 30–50-bp homology arms to the desired edit site, with mutations that were introduced to prevent Cas9 cleavage after the ssDNA donor was used for repair, and was synthesized as a 4 nmol Ultramer (IDT). All ssDNA oligos used for scarless recombineering are listed in Table S5. After transformation, cells were recovered at 30 °C for 2 h, before being plated on 25 μg/ml chloramphenicol, 200 μg/ml spectinomycin and 0.1 μg/ml anhydrotetracycline and incubated overnight. The next day, single colonies were checked by PCR and Sanger sequencing (Genewiz) to identify colonies with the desired edit. Then, the temperature sensitive gRNA expression plasmid was cured by growing cells at 42 °C. To cure the Cas9 plasmid, cells were made chemically competent and transformed with a Cas9-targeting sgRNA plasmid (pSL0407). After transformation, cells were recovered at 30 °C for 3 h, induced with anhydrotetracycline to 0.1 μg/ml, then continued recovery for an addition 2 h before plating on 200 μg/ml spectinomycin and 0.1 μg/ml anhydrotetracycline, and incubating at 30 °C overnight. The next day single colonies were grown up without antibiotics and the sgRNA plasmid was cured by overnight growth at 42 °C. Cultures were checked for curing of both Cas9 and sgRNA plasmid by spotting onto plates containing either chloramphenicol (25 μg/ml) or spectinomycin (200 μg/ml).

The original *Enterobacter sp.* BIDMC93 strain encodes three distinct flagellin genes: one located within the FRφ prophage (*fliC*_*P*_), one chromosomal copy that is targeted and silenced by TldR (*fliC*_*H*_), and a third, distinct chromosomal homolog that we refer to as *fliC*_*2*_. To assess the prevalence of this additional flagellin gene, we performed a systematic analysis of 396 genomic assemblies containing *fliC*_*P*_*–tldR*. Across these genomes, we identified 893 flagellin homologs. Our analysis revealed that an extra *fliC*_*2*_-like copy was present in only ~4.4% of *fliC*_*P*_*–tldR*–containing genomes, whereas a TldR-targeted *fliC*_*H*_-like homolog co-occurred with *fliC*_*P*_*–tldR* in approximately 93% of cases. Based on these findings, we concluded that *fliC*_*2*_ represents a rare and unregulated flagellin gene not integrated into the canonical TldR regulatory system. Therefore, to enable unambiguous functional analysis of FliC_P_ and FliC_H_, we deleted *fliC*_*2*_ from the *Enterobacter sp.* BIDMC93 background (*Enterobacter sp.* BIDMC93 *ΔfliC*_*2*_) and used this newly generated strain in all subsequent assays (see Table S4).

Plasmids used in this study are listed in Table S6. Flagellin expression vectors were constructed by cloning flagellin genes under a medium-strength constitutive promoter (J23105) on a medium-copy pET vector with carbenicillin resistance. TldR was cloned into expression vectors under the control of constitutive J23105 promoters, and guide RNAs were expressed using constitutive J23119 promoters. Derivatives of these expression plasmids were cloned using a combination of methods, including Gibson assembly, restriction digestion-ligation, and around-the-horn PCR. Plasmids were cloned, propagated in NEB Turbo cells, purified using Miniprep kits (QIAGEN), and verified by Sanger sequencing (Genewiz).

### Bacterial motility assays

Overnight cultures were diluted 1:100 in LB supplemented with antibiotic, then grown with gentle shaking (~120 rpm) to OD_600_ = 0.6 at 30 °C. Then, 2 μL of culture was pipetted onto the center of semisolid agar plates (2.5% Miller’s LB broth and 0.2% Bacto agar) and incubated at 30 °C before imaging. Images were captured on a Bio-Rad Gel Doc XR Imaging system using epi-illumination and automatic exposure. Halo size was measured by taking the average of the vertical and horizontal diameter measurements for three replicates.

### Automated growth curve measurements

Overnight cultures of the indicated strains were diluted 1:100 into 200 μL of LB supplemented with the appropriate antibiotics in a 96-well microplate. Cultures were grown in triplicate for 10 h at 37 °C in a BioTek Synergy Neo2 plate reader, with absorbance at 600 nm recorded every 10 min. Growth curves represent the mean of three biological replicates (n = 3), and shading indicates the standard deviation at each time point. Growth rates were calculated as the slope of the exponential growth phase (30 min to 2 h post-inoculation) following log_2_ transformation of the OD measurements. Growth rates were normalized to those obtained for the WT strain.

### Flagellar filament purification

Flagellar filaments were purified by mechanical shearing and centrifugation, essentially as previously described^52^. Overnight cultures of each *E. coli* strain (with constitutive episomal flagellin expression) were grown in 100 μg/ml carbenicillin with gentle shaking (~120 rpm) for 6 h. Cultures were then normalized by OD_600_ and centrifuged at 4,000 *g* for 5 min, then the pellet was resuspended in deflagellation buffer (1 M Tris-Cl (pH 6.5) and 100 mM NaCl). Flagellar filaments were mechanically sheared from cell bodies by passing cells through a 27-gauge needle 15–20 times, and the filament fraction was isolated from cell bodies by centrifugation at 10,000 *g* for 15 min. The supernatant containing flagellar filaments was removed and concentrated 1,000-fold through a 100-kDa filter (Amicon Ultra Centrifugal Filter). Then, the buffer was exchanged to motility buffer (0.5 mM CaCl_2_, 0.1 mM EDTA, 20 mM HEPES (pH 7.5)) via dialysis (Thermo Scientific Slide-A-Lyzer MINI Dialysis Unit).

### Cryogenic electron microscopy

3 μL of purified flagellin samples were applied to a glow-discharged holey carbon Quantifoil Au R1.2/1.3 grids (Electron Microscopy Science, Hatfield, PA), and blotted with filter paper using a FEI Vitrobot Mark III at 4 °C and 100% humidity (blot force = 0, and blot time = 3.0–4.5 s) then plunge-frozen into liquid ethane. Preliminary screening was performed in a Glacios instrument (ThermoFisher) to check for ice quality and sample concentration/quality. Grids showing good contrast and long and multiple filaments per hole were saved for high-resolution data collection on a Titan-Krios instrument equipped with a BioQuantum-K3 direct detector with an integrated energy filter. Movies were collected at a magnification of 105,000x corresponding to a pixel size of 0.844 Å. Exposure time and fluence were adjusted to ~1e^−^/pixel/frame and 60 frames per movie were collected with a defoci range of −0.8 to −2.2 μm for a total dose of 61.02 e^−^/Å^2^. Cryo-EM data collection and model refinement statistics are summarized in Table S8.

### Helical reconstruction and structure determination.

Cryo-EM data processing was integrally performed in Relion5^18^ with ctf estimation in CTFFIND4^53^ via the Relion wrapper. Motion correction was performed in whole frame mode and particle picking was performed with a modified version of Topaz that allows automatic filament picking^54^ . First, we selected 50 images of each dataset that were manually picked and extracted using canonical values of the *E. coli* flagellum structure as reference (*id est* 4.85 Å rise). Particle stacks were generated by sliding a box of 600 pixels 48.5 Å apart along the picked filament (10 times the canonical rise) and these particles were subsequently binned to a final size of 200 pixels. Two rounds of 2D classification (100 classes each, tau fuge parameter (T) 2 and 4) allowed the selection of 8 2D class averages showing secondary structure elements. This set of particles (~3000) was used for training a Topaz^55^ model for automatic filament picking of the whole dataset that produced a large dataset of 3 million particles. Recursive 2D classifications with increasing T values (from 2 to 5) and selecting for the best looking 2D averages in terms of secondary structure detail and straightness rendered a final set of ~125,000 particles. The Fourier transform of the final 2D averages revealed a pattern of layer lines reminiscent of previous studies of bacterial filaments by cryo-EM^52,54^. The presence of additional layer lines beyond the canonical helical core of the *E. coli* flagellum hinted towards multiple symmetries in the same filament. To explore this possibility, we created a cylindrical mask including the external domains of the filaments but excluding the canonical helical core formed by the coiled-coils encoded in the D0-D1 domains. This approach allowed us to eliminate (using the signal subtraction feature of Relion5^18^) the signal corresponding to the central helical core and reconstruct using helical reconstruction without symmetry, the signal corresponding to the external sheath using as initial reference a smooth cylinder^18,56^ . This approach rendered low resolution (~8 Å) but highly informative reconstructions whose interpretation was facilitated by the AlphaFold 3 (AF3) predictions of the monomer structures for *Enterobacter* FliC_H_ and FliC_P_. By manually docking of the AF3 prediction for the external domains of FliC_H_ in the low-resolution maps obtained by helical reconstruction without symmetrical impositions, we were able to recognize a unit of 5 consecutive monomers that seemed to be repeating along the filament. This observation prompted us to attempt a helical reconstruction of the whole filament using 5 times the canonical values of the *E. coli* central core, that is rise of 24.4 Å and twist of -33.5 º (canonical values are rise 4.88 Å and twist 65.4 °). This refinement converged to a map of around 4 Å with correct features for a map of this resolution such as well defined secondary structure elements including β-strand separation and identifiable bulky side chains. Ctf-parameters refinement and estimation of local particle movement and signal filtering using the Relion5 bayesian polishing approach rendered a final map with overall resolution of 3.7 Å^57^ . The final map for FliC_H_ shows excellent density for the canonical core including many side chains as well as unambiguous density for the external domains.

For the FliC_P_ case we applied a similar strategy, isolating via signal subtraction the external layer of the filament and performing a helical reconstruction without symmetry. The interpretation of this map revealed a nuanced pattern of interactions whose interpretation was possible using the AF3 prediction for the FliC_P_ monomer. The FliC_P_ monomer adopts two conformations that we termed “up” and “down” which differ in a close to 180 ° flip of the D2-D3-D4 domains relative to the canonical D0-D1 domains (similar to previously reported flagellum structures from soil bacteria^52^ ). One monomer in “down” conformation interacts with another monomer in “up” conformation located in an adjacent protofilament, creating a dimeric unit that is repeated. Along a vertical protofilament, the repetition pattern is “down-down-up” and “up-up-down” in the neighboring protofilament. However, this pattern is not compatible with the underlying canonical symmetry of the D0-D1 domains when considering the eleven protofilaments that constitute the entire flagellum. This inconsistency is solved by alteration of the “down-down-up” pattern introducing a symmetry disruption. To generate a map of the complete filament for the FliC_P_ we improved the resolution of the external sheltering layer by ctf-parameter refinement and bayesian polishing to ~7 Å reconstructing helically but without imposing any symmetry. In parallel, we refined the helical core to ~3.5 Å applying helical reconstruction and the canonical flagellum parameters (rise 4.88 Å and twist 65.4 °). We produced a composite map aligning the N-terminal helix of D1, visible in the external layer map and merging both maps to a final resolution of 7 Å.

For model building of FliC_H_, using UCSF Chimera^58^, we manually placed 88 monomers in the final unsharpened map, carefully adjusting the position of the D2-D3 external domains relative to the D0-D1 canonical domains for every monomer. We then divided the fitted monomers into vertically aligned groups of 11 protofilaments and every proto-filament was stereochemically refined in real-space against the post-processed map using phenix-real-space refinement with secondary structure restraints activated^59^ . Following this step, we performed a reciprocal-space refinement for every protofilament using REFMAC5^60^ with secondary structure restraints calculated in ProSMART^61^.

For FliC_P_ we employed a similar model-building approach but due to the limited resolution we restricted our model refinement to a single step of real-space refinement in phenix using tight secondary structure restraints to avoid overfitting of the atomic model.

### Flagellin imaging

TC-tagged flagella were labeled using FlAsH, essentially as previously described^62^, with some modifications. This protocol was adapted to label AviTag flagella using streptavidin-Alexa Fluor. Overnight cultures were diluted 1:100 in LB supplemented with antibiotic, and grown with gentle shaking (~120 rpm) for 6 h. Cells were then labeled by incubating them with the appropriate dye in LB. For labeling TC-tagged flagella, cells were labeled with 2.5 μM FlAsH (Thermo Fisher) and incubated at 30 °C for 2 h. For labeling AviTagged flagella, cells were labeled with 10 μg/ml streptavidin-Alexa Fluor 568 conjugate (Invitrogen) and incubated at 30 °C for 2 h. For co-labeling cells, both 2.5 μM FlAsH and 10 μg/ml streptavidin-Alexa Fluor 568 were added to cells.

Microscopy tunnel slides were prepared using two pieces of double-sided tape between microscope slides (Fisherbrand, 75 × 25 mm) and a coverslip (Fisherbrand, 22 mm, No 1.5). The tunnel was treated with 25 μL of 0.1% poly-L-lysine (Sigma) for 5 mins, then washed with M9 media. 20 μL of labeled cells were pipetted into the tunnel and allowed to sit for 10 mins, to adhere to the coverslip, before being washed twice with 50 μL M9 to remove unadhered cells. Images were captured using a Zeiss Axiovert 200 inverted microscope equipped with a 100X oil objective and an Axiocam 506 monochrome camera. Images were pseudo-colored using ImageJ^63^ when merging two channels.

### Chromatin-immunoprecipitation followed by next generation sequencing (ChIP-seq)

Cells were scraped from solid agar plates and resuspended in 40 ml of LB media. Cells were fixed by adding formaldehyde to final concentration of 1%, and nutated for 20 min at room temperature. Formaldehyde was quenched by adding 4.6 ml of 2.5 M glycine and nutating for 10 min. Cells were then centrifuged at 4000 *g* at 4 °C for 8 min, and the pellet was washed twice in cold 1× TBS. To normalize cell density across cultures, the equivalent of 40 ml of OD_600_=0.6 was transferred to a tube, and pelleted again by centrifugation. The pellet was then transferred to a 1.5 ml Eppendorf tube and centrifuged at 10,000 *g* at 4 °C for 5 min. The supernatant was discarded and pellets were flash-frozen in liquid nitrogen.

Frozen cell pellets were resuspended in lysis buffer (50 mM HEPES-KOH (pH 7.5), 0.1% (w/v) sodium deoxycholate, 0.1% (w/v) SDS, 1 mM EDTA, 1% (v/v) Triton X-100, 150mM NaCl, 1× cOmplete Roche protease inhibitor), and transferred to a 1 ml milliTUBE AFA Fiber (Covaris). Cells were sonicated on an M220 Focused-ultrasonicator (Covaris) with the following SonoLab 7.2 settings: minimum temperature, 4 °C; set point, 6 °C; maximum temperature, 8 °C; peak power, 75.0; duty factor, 10; cycles/bursts, 200; sonication time, 17.5 min. After sonication, 10 μL of cleared lysate was withdrawn and kept as the pre-IP control (“input”). The remainder was used for immunoprecipitation.

For immunoprecipitation, 25 μL Dynabeads Protein G (Thermo Fisher Scientific) for each sample was washed four times in 1× PBS + 0.5% BSA, then mixed with 4 μL monoclonal anti-FLAG M2 antibody produced in mouse (Sigma-Aldrich F1804), which was conjugated at 4 °C for 6 h. After conjugation, beads were washed four times with 1× PBS + 0.5% BSA, resuspended in 30 μL lysis buffer, then mixed with sonicated samples and rotated at 4 °C overnight.

The following day, beads were washed with lysis buffer lacking protease inhibitor three times, then washed once with lysis buffer lacking protease inhibitor and supplemented with 500 mM NaCl, once with ChIP wash buffer (10 mM Tris–HCl (pH 8.0), 250 mM LiCl, 0.5% (w/v) sodium deoxycholate, 0.5% (v/v) Nonidet-P40, 1 mM EDTA) and finally twice with TE buffer (10 mM Tris-HCl (pH 8.0), 1 mM EDTA). After the final wash, beads were resuspended in 200 μL elution buffer (1% (w/v) SDS, 0.1 M NaHCO_3_) and incubated at 65 °C for 1 h 15 min to release protein-DNA complexes from the beads, vortexing every 15 min to resuspend the beads. While incubating the immunoprecipitated samples, 10 μL of the frozen pre-IP samples were withdrawn and 190 μL elution buffer was added. After the 65 °C incubation was complete, 10 μL of 5 M NaCl was added to each pre-IP and IP sample, which were incubated at 65 °C overnight to reverse crosslinks.

After the overnight incubation, RNA digestion was performed using 1 μL RNase A (Thermo Fisher Scientific) and incubating at 37 °C for 1 h. Protein digestion was performed using 2.8 μL of 20 mg/ml proteinase K (Thermo Fisher Scientific) and incubating samples at 55 °C for 1 h. DNA was purified (QIAquick PCR Purification Kit) and eluted in 40 μL TE buffer before the concentration was measured by fluorometry (DeNovix dsDNA Ultra High Sensitivity Kit). Samples were normalized to the lowest concentration, and DNA was prepared for next-generation sequencing using the NEBNext Ultra II DNA Library Prep Kit for Illumina (NEB). Samples were sequenced using an AVITI Sequencing System with a 2×150 Cloudbreak Freestyle Kit (Element Biosciences).

After sequencing, paired-end reads were filtered and trimmed using fastp^64^ and mapped to the *E. coli* MG1655 or *Ent* reference genome using bowtie2. Then, we used Samtools to sort, index, and filter multi-mapping reads. Finally, coverage was normalized to counts per million (CPM) using the deepTools2 command bamCoverage (v3.5.1).

### RNA immunoprecipitation (RIP-seq) of RNA bound by TldR

Cells harvested for RIP-seq were cultured as described for ChIP-seq using an *E. coli* MG1655 strain. Cells were scraped from solid agar plates and resuspended in 1 ml of TBS buffer (20 mM Tris-HCl pH 7.5, 0.15 M NaCl). Next, cell density was measured (OD_600_) and an equivalent of 20 ml of OD_600_=0.5 was withdrawn into a new tube. Cells were pelleted by centrifugation at 4,000 *g* and 4 °C for 5 min, the supernatant was discarded, and pellets were flash frozen in liquid nitrogen.

Cell pellets were resuspended in 1.2 ml RIP lysis buffer supplemented with cOmplete Protease Inhibitor Cocktail (Roche) and SUPERase•In RNase Inhibitor (Thermo Fisher Scientific). Cells were then sonicated for 1.5 min total (2 sec ON, 5 sec OFF) at 20% amplitude. Lysates were centrifuged for 15 min at 4 °C at 21,000 *g* and the supernatant was transferred to a new tube. At this point, a small volume of each sample (24 μl, or 2%) was set aside as the input material.

For immunoprecipitation, 60 μl Dynabeads Protein G (Thermo Fisher Scientific) were first washed 3 times in 1 ml RIP lysis buffer (20 mM Tris-HCl pH 7.5, 150 mM KCl, 1 mM MgCl_2_, 0.2% Triton X-100), then resuspended in 1 ml RIP lysis buffer and combined with 20 μl anti-FLAG M2 antibody (Sigma-Aldrich). Samples were rotated at 4 °C for at least 3 h. Antibody-bead complexes were washed 3 times and resuspended in 60 μl RIP lysis buffer, then mixed with sonicated samples and rotated at 4 °C overnight.

The following day, samples were washed three times with ice-cold RIP wash buffer (20 mM Tris-HCl, 150 mM KCl, 1 mM MgCl_2_). After the final wash, beads were resuspended in 1 ml TRIzol (ThermoFisher Scientific) and RNA was eluted from the beads by incubating at RT for 5 min. The supernatant was separated using a magnetic rack and transferred to a new tube, then combined with 200 μl chloroform. Each sample was mixed vigorously by inversion, incubated at RT for 3 min, and centrifuged for 15 min at 4 °C at 12,000 *g*. RNA was isolated from the upper aqueous phase using an RNA Clean & Concentrator kit (Zymo). RNA from input samples was isolated in the same manner, using TRIzol and column purification. High-throughput sequencing library preparation was per-formed as described below for total RNA-seq of *Enterobacter* strains. Libraries were sequenced on an AVITI Sequencing System with a 2×150 Cloudbreak Freestyle Kit (Element Biosciences).

Adapter trimming, quality trimming, and read length filtering of RIP-seq reads was performed as described below for total RNA-seq experiments. Trimmed and filtered reads were mapped to the *E. coli* MG1655 reference genome using bwa-mem2v2.2.1, with default parameters. Mapped reads were sorted, indexed, and converted into coverage tracks as described below for total RNA-seq experiments.

### Total RNA sequencing of *Enterobacter*

For RNA-sequencing on *E. coli* WT and the two RNase III knockout strains (*Δrnc* and *rnc105*), colonies were first scraped from solid media, resuspended in LB media, and the equivalent of 1 mL of OD_600_=1 culture was withdrawn. Samples were centrifuged at 3,000 *g* at 4 °C for 5 min, then the supernatant was discarded. RNA was extracted by adding 750 μL TRIzol, incubating for 5 min at room temperature, then 150 μL chloroform was added to each tube, shaken vigorously to mix, and left for 5 min at room temperature. Samples were centrifuged at 12,000 *g* at 4 °C for 15 min, then 400 μL of the upper aqueous layer was carefully transferred to a new tube. RNA was purified using an RNA Clean and Concentrator Kit (Zymo), eluting in 15 μL of DNase/RNase-free water. Samples were then diluted in NEBuffer 2 (NEB) to a total volume of 15 μL, and fragmented by incubating at 92 °C for 2 min. The fragmented RNA was simultaneously treated with RppH (NEB) and TURBO DNase (Thermo Fisher Scientific) in the presence of SUPERase•In RNase Inhibitor (Thermo Fisher Scientific) at 37 °C for 30 min, in order to remove DNA and 5′-pyrophosphate. For end repair, the RNA was treated with T4 PNK (NEB) in 1×T4 DNA ligase buffer (NEB), and incubated at 37 °C for 30 min. Samples were column-purified using an RNA Clean & Concentrator Kit (Zymo), and the concentration was determined using the DeNovix RNA Assay (DeNovix). Illumina adapter ligation and cDNA synthesis were performed using the NEBNext Small RNA Library Prep kit, using 100 ng of RNA per sample. High-throughput sequencing was performed on an AVITI Sequencing System with a 2×150 Cloudbreak Freestyle Kit (Element Biosciences).

RNA-seq reads were processed using cutadapt (v4.2) to remove adapter sequences, trim low-quality ends from reads, and exclude reads shorter than 15 bp. Trimmed and filtered reads were aligned to the MG1655 reference genome using bwa-mem2 (v2.2.1) in paired-end mode with default parameters. SAMtools (v1.17) was used to filter for uniquely mapping reads using a MAPQ score threshold of 1, and to sort and index the unique reads. Coverage tracks were generated using bamCoverage (v3.5.1) with a bin size of 1, read extension to fragment size, and normalization by counts per million mapped reads (CPM) with exact scaling. Coverage tracks were visualized using IGV.

### *Enterobacter* phage induction and isolation

Phage induction was performed using mitomycin C treatment, essentially as previously described^65^ with minor modifications. Overnight cultures of *Enterobacter* sp. BIDMC93 (*Ent*) strains were diluted 1:100 in LB media, and incubated at 37 °C with agitation until OD_600_=0.20. Then, cultures were treated with final concentration of 0.5 μg/ml mitomycin C and incubated at 37 °C for 16 h. Chloroform was added to a final concentration of 4% (v/v) and briefly vortexed to facilitate bacterial lysis, after which the lysate was centrifuged at 4000×*g* for 10 min to pellet cell debris. The supernatant was carefully withdrawn, taking care not to disrupt the chloroform-cell interface, and passed through a 0.22 μm filter. The phage-containing filtrate was stored at 4 °C.

### Lysogenization assay by dual-antibiotic resistance screening

To detect lysogenization, we used a custom assay that relied on labeling phage and bacteria with separate antibiotics and detecting cells that had acquired resistance to both antibiotics after mixing cells with phage particles. We used λ-Red recombineering (as described above) to label the prophage with a chloramphenicol resistance cassette and the *Enterobacter* genome with a kanamycin resistance cassette. After inducing cells with mitomycin C and isolating phage-containing filtrate by sterile filtration (described above), 450 μL of recipient cells at OD_600_=0.20 were mixed with 50 μL of isolated phage particles, then incubated with shaking at 37 °C for 2 h before plating. Cells were serially diluted (10×) and plated as 4 μL spots onto LB agar supplemented with either single antibiotics (50 μg/ml kanamycin or 25 μg/ml chloramphenicol) or double antibiotic plates (50 μg/ml kanamycin plus 25 μg/ml chloramphenicol). Plates were incubated at 37 °C for 16 h to allow the growth of colonies. Plates were imaged on a BioRad Gel Doc XR+ imager.

### Small drop plaque assays

Plaque assays were performed as previously described^66^. An overnight culture for each strain was mixed with 4 ml of freshly prepared molten soft agar (0.5% Difco agar + 2.5 % LB Miller’s broth) and poured over solid bottom agar (1.5 % agar + 2.5 % LB Miller’s broth, supplemented with the appropriate antibiotic) and rotated to evenly dispense the soft agar solution. The soft agar was left to solidify for 15 min at RT, during which 10× serial dilutions of phage samples were prepared in LB media. For plating, 3 μL of each phage dilution was spotted onto the surface of the soft agar, and plates were left to dry for 10 min under a laminar flow hood. Plates were incubated at 37 °C for 12–16 h to allow the formation of plaques. Plates were imaged on a BioRad Gel Doc XR+ imager.

### Isolation of FRφ-resistant mutants

*Enterobacter* cells with mutations in the FRφ receptor were isolated by performing whole-genome sequencing cells that grew after infection with lytic phage, essentially as previously described^67^ . Overnight cultures of *Enterobacter Δprophage* cells were diluted 1:100 in LB media supplemented with 25 μg/ml chloramphenicol and incubated at 37 °C until OD_600_=0.3. 1 ml of bacterial culture was mixed with 1 ml of lytic FRφ (Δ*cI* or Δ*cII*, constructed using λ-Red recombineering, as described above). This mixture was incubated at 37 °C for 10 min, then plated on solid LB agar plates supplemented with 25 μg/ml chloramphenicol, which were left overnight at 37 °C.

The next day, individual colonies were grown in LB media and checked by plaque spot assay (described above) to confirm resistance to FRφ. To identify mutations conferring resistance, cultures were sent for long-read whole genome sequencing (performed by Plasmidsaurus using Oxford Nanopore Technology with custom analysis and annotation). Annotated genomes generated from long-read sequencing data were analyzed for single nucleotide polymorphisms (SNPs) using Parsnp2. This analysis revealed mutations in FhuA or FhuB. Mutations in FhuA were confirmed by rescue experiments, in which *Enterobacter* FhuA and its native promoter was cloned onto a plasmid (pSL7573), and used for transformation into *Enterobacter ΔfhuA* cells. Plaquing assays (described above) performed using this strain confirmed that expression of FhuA rescued the ability of FRφ to infect and lyse cells.

### Screening wastewater for flagellotropic *Enterobacter* phage

We screened wastewater for flagellotropic *Enterobacter* phage using two alternative methods, referred to as replica-plaquing and using the previously described Phage DisCo approach^26^. For the replica-plaquing approach, we obtained wastewater from Bozeman (Montana), New York City (New York) and Boston (Massachusetts). The viral fraction of each sample was obtained by treatment with 4% chloroform, vortexing, spinning and passing the aqueous phase through a 0.22 μm filter. 200 ml of the wastewater viral fractions obtained in Bozeman and New York were then concentrated 100-fold by passing them through a 50 kDa filter to a final volume of 2 ml. The Boston viral suspension and the concentrated Bozeman and New York City samples were then used to obtain plates on an *Enterobacter Δprophage* lawn. Briefly, 100 μL of each sample were mixed with 100 μL of an exponentially growing culture of *Enterobacter Δprophage* at OD ~ 0.3, incubated at room temperature for 5 minutes, then mixed into 4 ml of freshly prepared molten soft agar supplemented with 5 mM MgCl_2_. The suspension was briefly inverted three times to mix, then poured over solid bottom agar (1.5 % agar + 2.5 % LB Miller’s broth, supplemented with the appropriate antibiotic). The soft agar was left to solidify for 15 min at RT and the obtained double layer agar plates were placed in a 37 °C incubator overnight. The following day, putative plaques were isolated using a 200 μL pipette tip and resuspended in 200 μL magnesium saline buffer (100 mM NaCl, 8 mM MgSO4 and 50 mM Tris at pH 7.5). Plaques isolated in this way are very small (< 1 mm) and may be confused with debris or bubbles in the soft agar. Therefore, to confirm that they can in turn form a larger plaque on a bacterial lawn, 3 μL of each resuspended putative plaque was pipetted onto a double-layer agar plate prepared with the *Enterobacter Δprophage* strain. Of the 868 putative plaques, 632 were confirmed as forming real plaques in this way. The corresponding plaques were isolated in 96-well plates, and the phage suspensions were treated with chloroform in a spin plate, wrung out and 100 μL of the supernatant pipetted into a new plate for storage at 4°C. The screen then consisted in stamping the contents of each plate, using a 96-pin replicator, onto double-layer agar plates containing either *Enterobacter Δprophage* or *Enterobacter Δprophage ΔfliC*_*H*_, in order to identify plaques that would form on the former but not on the latter. Of all the pages tested in this way, no phage exhibited this characteristic.

Phage DisCo was performed essentially as previously described^26^, with minor modifications. An equimolar ratio of mid-log phase cells for *Enterobacter Δprophage* cells expressing RFP under a constitutive promoter (J23119) were mixed with Δ*fliC*_*H*_ *Δprophage* cells expressing GFP under a constitutive promoter (J23105). A total of 100 μL of mixed strains plus 100 μL of phage suspension isolated from environmental samples were mixed with 4 ml of freshly prepared molten soft agar (0.5% Difco agar + 2.5 % LB Miller’s broth). The suspension was briefly inverted three times to mix, then poured over solid bottom agar (1.5 % agar + 2.5 % LB Miller’s broth, supplemented with the appropriate antibiotic) and rotated to evenly dispense the molten agar solution. The soft agar was left to solidify for 15 min at RT before placed in a 37 °C incubator overnight. The following day, plates were imaged on Amersham Typhoon Biomolecular imager (Cytiva) using 488 nm and 635 nm laser intensities. Images were pseudo-colored using ImageJ when merging two channels.

### TLR5/NF-κB Assay in HEK293T-Blue-hTLR5 cells.

HEK293T-Blue-hTLR5 are HEK293T cells co transfected with the human TLR5 gene and an inducible SEAP (secreted embryonic alkaline phosphatase) reporter (Invivogen, HKB-HTLR5). This cell line is not part of the list of known misidentified cell lines maintained by the International Cell Line Authentication Committee (https://iclac.org/databases/cross-contaminations/). The SEAP gene is placed under the control of the IFN-β minimal promoter fused to five NF-κB and AP-1-binding sites. Stimulation with a TLR5 ligand (i.e. flagellin) activates NF-κB and AP-1 which induces the production of SEAP. This enzyme induces a color shift from pink to blue of the chromogenic substrate in the HEK-Blue Detection Medium (Invivogen). TLR5 activation can then be measured by colorimetry at OD 655 nm. The cell line was cultured at 37 °C and 5% CO_2_ and maintained in DMEM media with 10% FBS and 100 U ml^−1^ of penicillin and streptomycin (Thermo Fisher Scientific), and 100 μg/mL Zeocin. The TLR5 activation assays were performed as follows:

Bacterial strains were cultivated for about 2 h with agitation in LB supplemented with the appropriate antibiotics. When all the strains reached OD_600_ ~ 0.3–0.4, they were all normalized to OD = 0.3. Aliquots of the OD-normalized culture were serially diluted and plated for CFU measurements. Other aliquots of the OD-normalized culture were diluted 100 times in PBS and 20 μL of resuspended bacterial cells were pipetted into a 96-well plate. 180 μL of HEK293T-Blue-hTLR5 resuspended in HEK-Blue Detection Medium were then added on top of the agonists in each well of the plate (1.4 × 10^5^ cells/mL, corresponding to 25000 cells per well and a MOI~20–30). Plates were incubated for 16h to 24h at 37°C, 5% CO_2_. In each well, the activation of NF-κB through TLR5 was assessed by absorbance measurements at 630 nm. Signal was then normalized according to CFU/mL and expressed as a % from the reference as indicated in each graph.

### Mice gut colonization experiment.

All mouse procedures were conducted following the guidelines of the Institutional Animal Care and Use Committee (IACUC) of Columbia University (Protocol: AABQ5551). Mice used in this study, strain C57BL/6, provided by the Jackson Laboratory, were 6 weeks old at the beginning of the experiment. Mice (all females) were housed at Columbia University Irving Medical Center, in a rodent pathogen-free environment, with a 12-hour light–dark cycle and provided ad libitum access to food and water. Housing conditions were maintained at 21–24 °C and 40–60% humidity. Sample size was determined on the basis of our previous studies and/or pilot experiments.

Two separate cohorts of 5 mice were used in this study, one gavaged with *Ent Δint* (deletion of FRφ integrase to ensure the prophage cannot get excised and lost during the assay) and the other gavaged with *Ent* Δprophage. Mice were gavaged on three consecutive days (24h between each gavage). For the gavage step, each strain was cultivated in 150 ml LB to OD ~ 0.3 and then renormalized to OD = 0.3. Cultures were centrifuged at 4000 *g* for 15 min and resuspended in 20 ml cold PBS in a 50 ml conical tube. They were centrifuged again at 4000 *g* for 15 minutes and resuspended in 2 ml cold PBS. The resulting suspension was washed once more in cold PBS and an aliquot was taken to assess CFU/mL (~ 3 × 10^10^ CFU/mL averaged over the three gavage steps). The remaining cell suspension was used to gavage the mice, with 200 μL (~ 6 × 10^9^ CFU/mL) of each strain administered to each mouse in the corresponding cohort. Faeces from each mouse in each cohort were collected (~50 mg) before the first gavage, after the first gavage and after the last gavage at the following times: 8 hours, 1 day, 2 days, 3 days, 4 days, 5 days, 7 days, 10 days, 13 days and 25 days. Feces were weighed and resuspended in 200 μL PBS. Resuspended feces were spun down for 2 sec to pellet large dietary debris and the supernatant was serially diluted for plating. 100 μL of resuspended feces were plated on LB-Agar plates containing the appropriate antibiotic as well as serially diluted samples (ranging from 10^−1^ to 10^−4^) for each mouse of each cohort. Plates were incubated at 37 °C overnight and CFU were counted. CFU count was converted to CFU/mL and normalized by the corresponding feces mass resuspended in LB for each mouse of each cohort (CFU/(mL⋅mg)).

### Biofilm formation assay

Biofilm formation was assessed using a crystal violet (CV) staining assay, as previously described^68^ . Overnight bacterial cultures were diluted 1:100 in LB supplemented with the appropriate antibiotics and grown at 30 °C with agitation until mid-log phase (OD_600_ = 0.3). For each strain, 150 μL of culture was transferred into individual wells of a sterile, flat-bottom 96-well polystyrene plate (Corning). Plates were incubated statically at 30 °C for 72 h to allow biofilm formation. Following incubation, planktonic cells were removed by inversion, then rinsed twice by submerging in sterile water. Wells were stained with 175 μL of 0.1% (w/v) crystal violet solution for 10 min at room temperature. Unbound dye was removed by inverting the plate, and wells were rinsed three times with water to remove residual stain. Plates were patted dry and air-dried for 2 h until no visible moisture remained. The retained dye was solubilized with 200 μL of 30% (v/v) acetic acid for 10 min, and 150 μL of the resulting solution was transferred to a fresh 96-well clear plate for quantification. Absorbance was measured at 550 nm using a BioTek Synergy Neo2 plate reader. Biofilm formation was expressed as the mean ± s.d. of three biological replicates.

### Bioinformatic analyses of the FRφ and *Citrobacter* prophage tail fiber shufflons

To assess the recombination frequencies of FRφ tail fiber genes, high-throughput DNA sequencing of three FRφ samples was conducted by Plasmidsaurus. One sample was genomic DNA extracted from *Ent* encoding FRφ with a synthetic *cmR* insertion. Another sample was also from isolated *Ent* genomic DNA, with a *Δgin* mutation in FRφ. The third sample was genomic DNA isolated from wild type FRφ virions. FASTQ files obtained from Plasmidsaurus were imported into R, and the Biostrings package was used to probe for DNA sequencing reads that contained 25 bp of sequence at the 3′-end of the N-terminal region of the tail fiber gene concatenated to 25 bp of sequence from the 5′-end of each C-terminal region. Raw read counts corresponding to each species of tail fiber protein were graphed as a percentage of the total number of tail fiber reads using the ggplot2 package.

To assess recombination frequencies of the tail fiber shufflon from *Citrobacter amalonaticus*, three DNA sequencing datasets were downloaded from NCBI’s Short Read Archive database (SRR9220577, SRR9219931, and SRR9220572). The reads were pooled and used to construct a BLASTn database, which was then queried with the sequence of the tail fiber gene that was suspected to encode the N-terminus region (WP_192927590.1 from NZ_WWUN01000014.1). A single boundary demarcated the end of the N-terminal region, beyond which different C-terminal tail fiber genes were present. Reads that didn’t span this junction, or that lacked more than 15 nt of the C-terminal region, were filtered out. The remaining reads were then manually inspected to determine which species of C-terminal tail fiber gene was present, and raw read counts were graphed using the ggplot2 package.

### Phylogenetic analysis of *csrA*-associated TldR loci

To identify *csrA*-associated TldRs and their most closely related TnpB relatives, seven sequences previously reported to be *csrA*-associated TldRs (USF27889.1, WP_251316131.1, WP_204885655.1, WP_016321625.1, WP_016324391.1, WP_087343418.1, and WP_122790109.1) were used as queries for an online BLASTp search of the NCBI NR database [default parameters]. The top 100 hits from each BLAST search were pooled, resulting in 147 unique TldR sequences which were used to again query the NCBI NR database with BLASTp [-evalue 1e-50 -qcov_hsp_perc 80 -max_hsps 50 -max_target_seqs 500]. The resulting hits were filtered to remove TnpB/TldR sequences encoded within 20 kb of a contig end. Sequences were also clustered at an 80% amino acid identity threshold using CD-HIT, before cluster representatives were aligned with MAFFT (LINSI option). A tree was then built from the resulting alignment with FastTree (-wag -gamma options) and visualized with ITOL. Nuclease active site intactness was assessed by first identifying active site residues in one of the TnpB homologs (WP_087343418.1) via comparison to the ISDra2 TnpB sequence, then inspecting the multiple sequence alignment for conservation at the positions corresponding to those active site residues.

To assess genetic associations, loci were extracted from genomes encoding TnpB/TldR—comprising 20 kb of sequence flanking each *tnpB*/*tldR* gene. These loci were then annotated with Emapper from EggNog, and open reading frames (ORFs) annotated as *csrA* were extracted and translated. The CsrA ORFs were then searched against all the ORFs predicted in the *tnpB*/*tldR* loci via BLAST, to ensure that none were missed or misannotated. A similar process was carried out with ORFs annotated as Flagellin, which are referred to as *fliC* for consistency, but are also denoted as *hag* in Firmicutes. TnpB/TldR homologs encoded within 3 kb of *csrA* or *fliC* were annotated as genetically associated with those genes.

### Phylogenetic analysis of Gin proteins

To identify homologs of the *Ent* Gin protein, Gin was used to query an online BLASTp search of the NCBI Clustered_nr database [default parameters]. The top 1000 hits from the BLAST search were clustered at a 90% identity threshold using CD-HIT, and then filtered to only include loci proteins encoded within loci that were at least 4-kb length. The Gin protein sequences were then aligned with MAFFT, and a tree was built from the resulting alignment using FastTree (-wag -gamma options), which was visualized in ITOL. Tn3 associations were assessed via tBLASTn searches of *gin* loci, and by searching ORFs predicted in *gin* loci [getorf function of EMBOSS; -table 1] with a profile HMM built from Tn3-like transposase sequences (PFAM: PF01526).

## Extended Data

**Figure:**
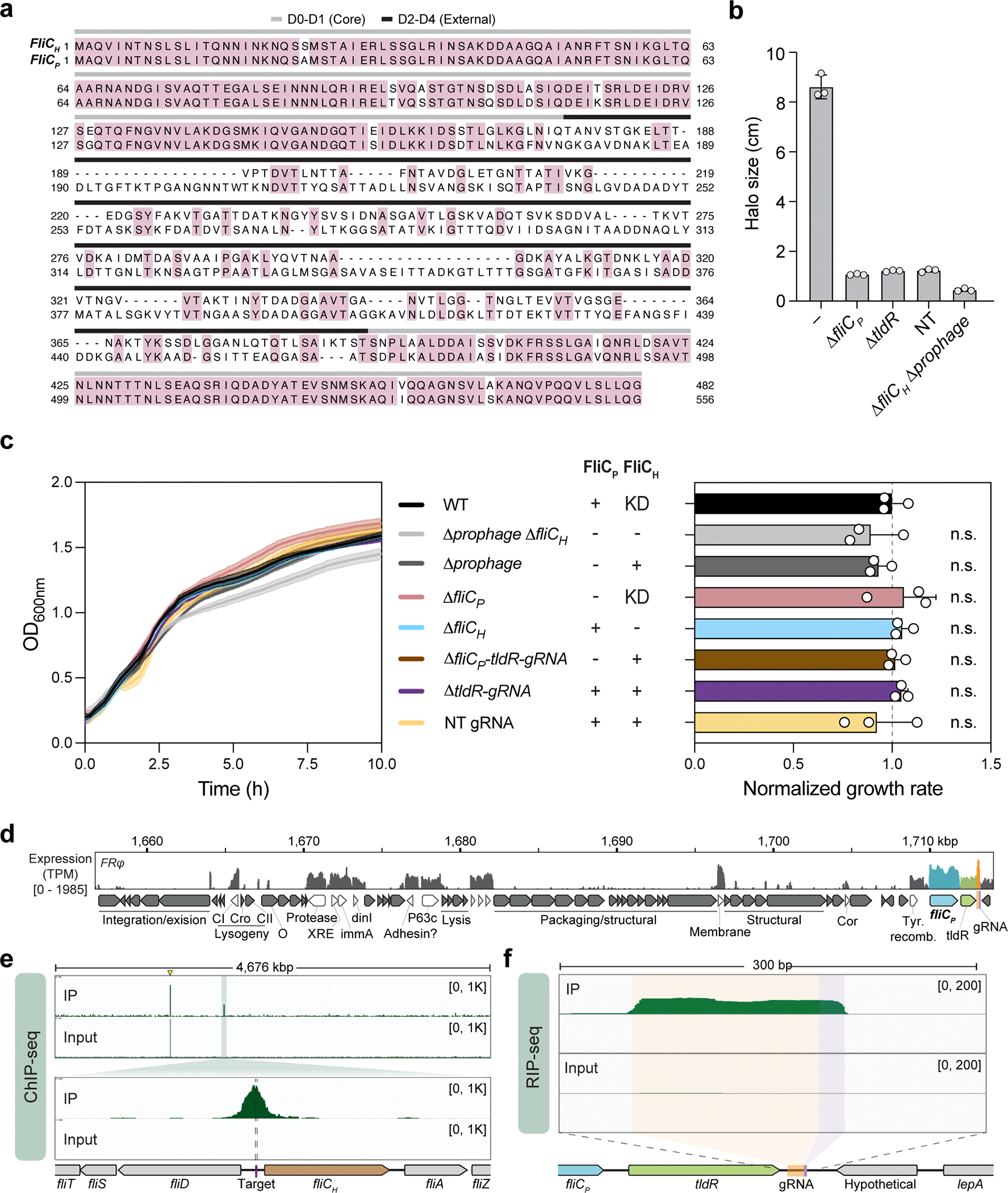


**Figure:**
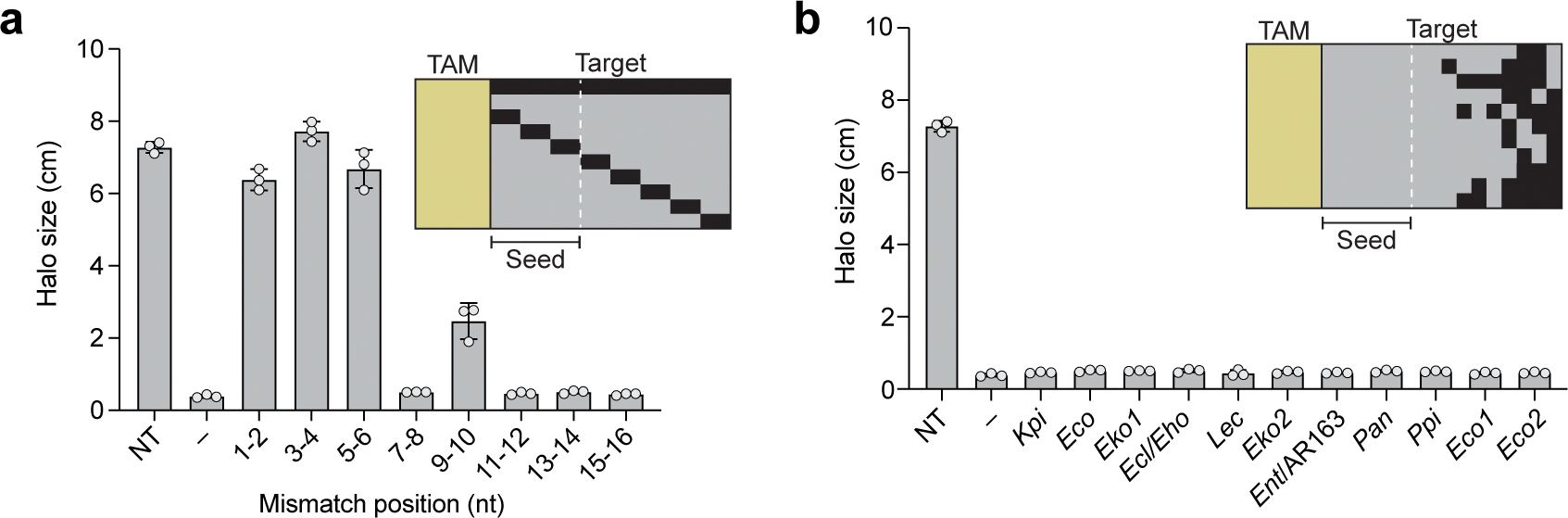


**Figure:**
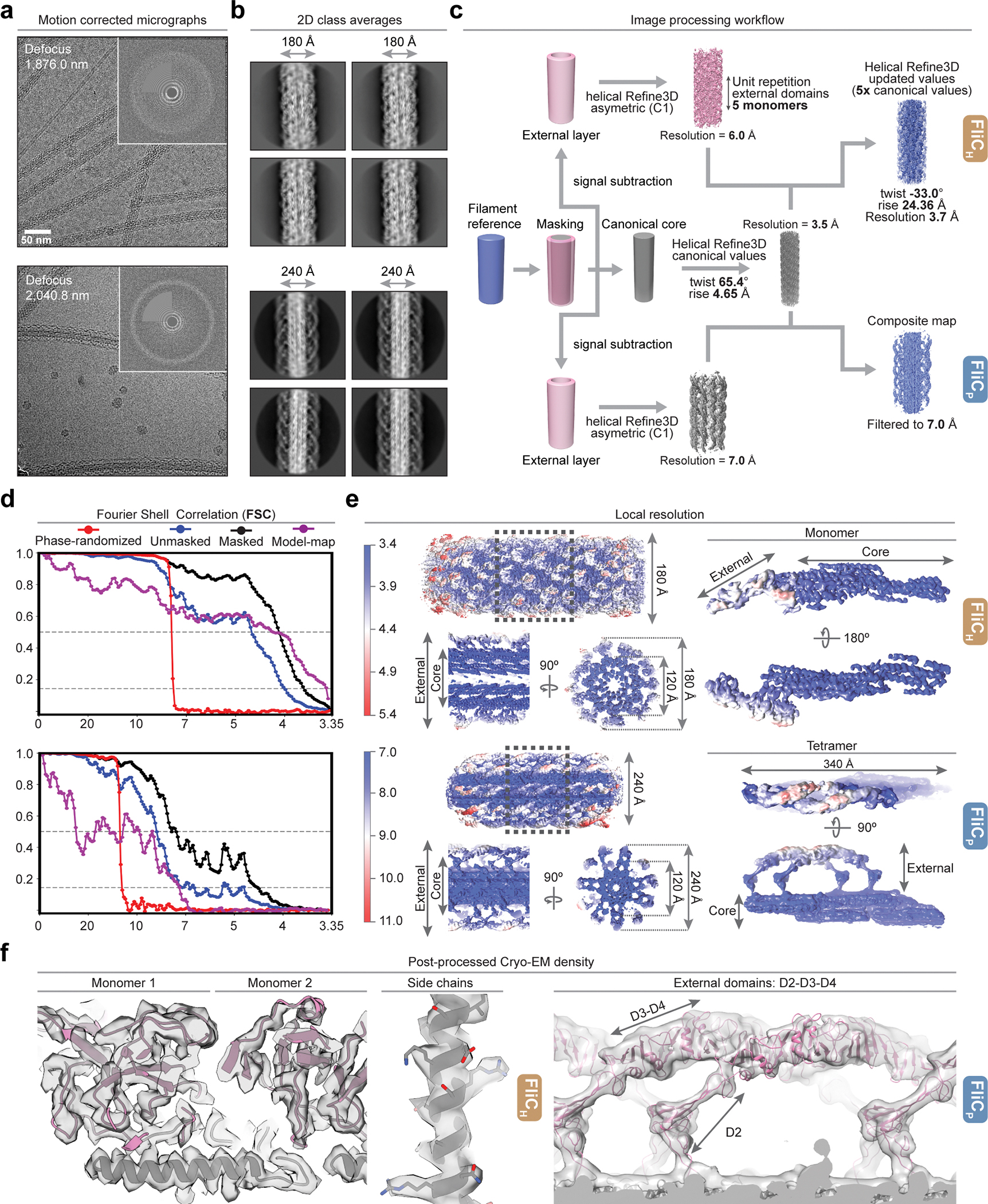


**Figure:**
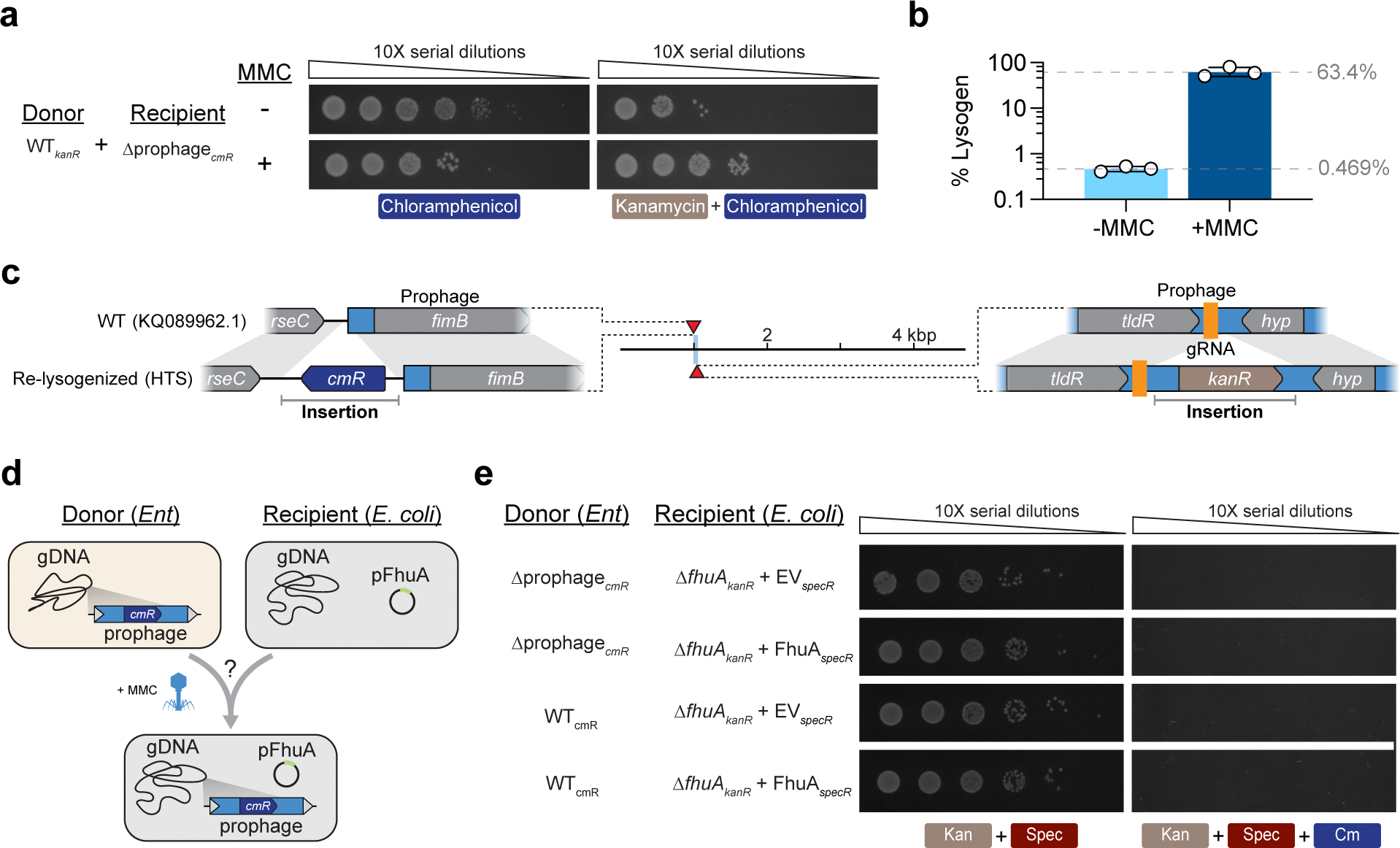


**Figure:**
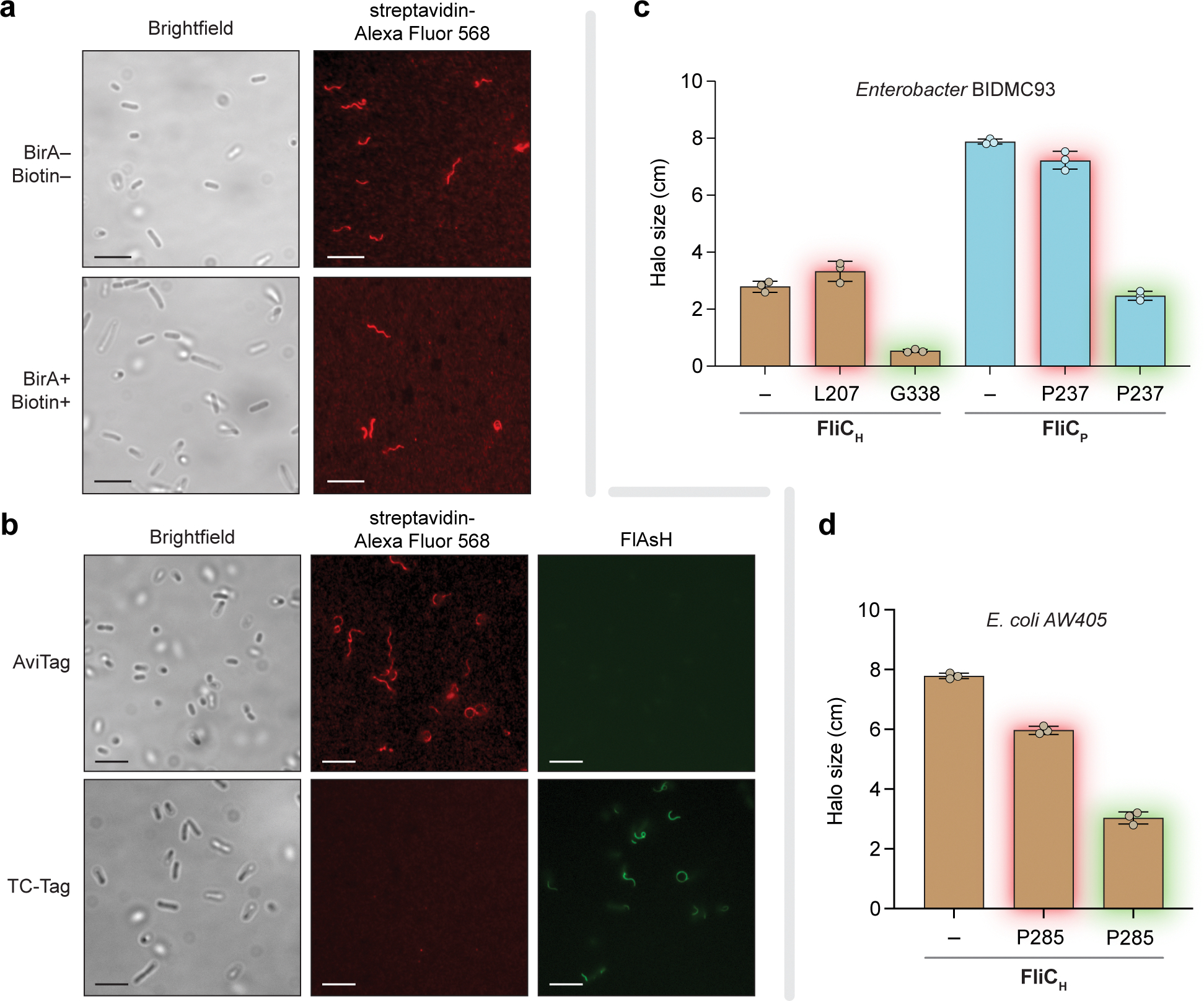


**Figure:**
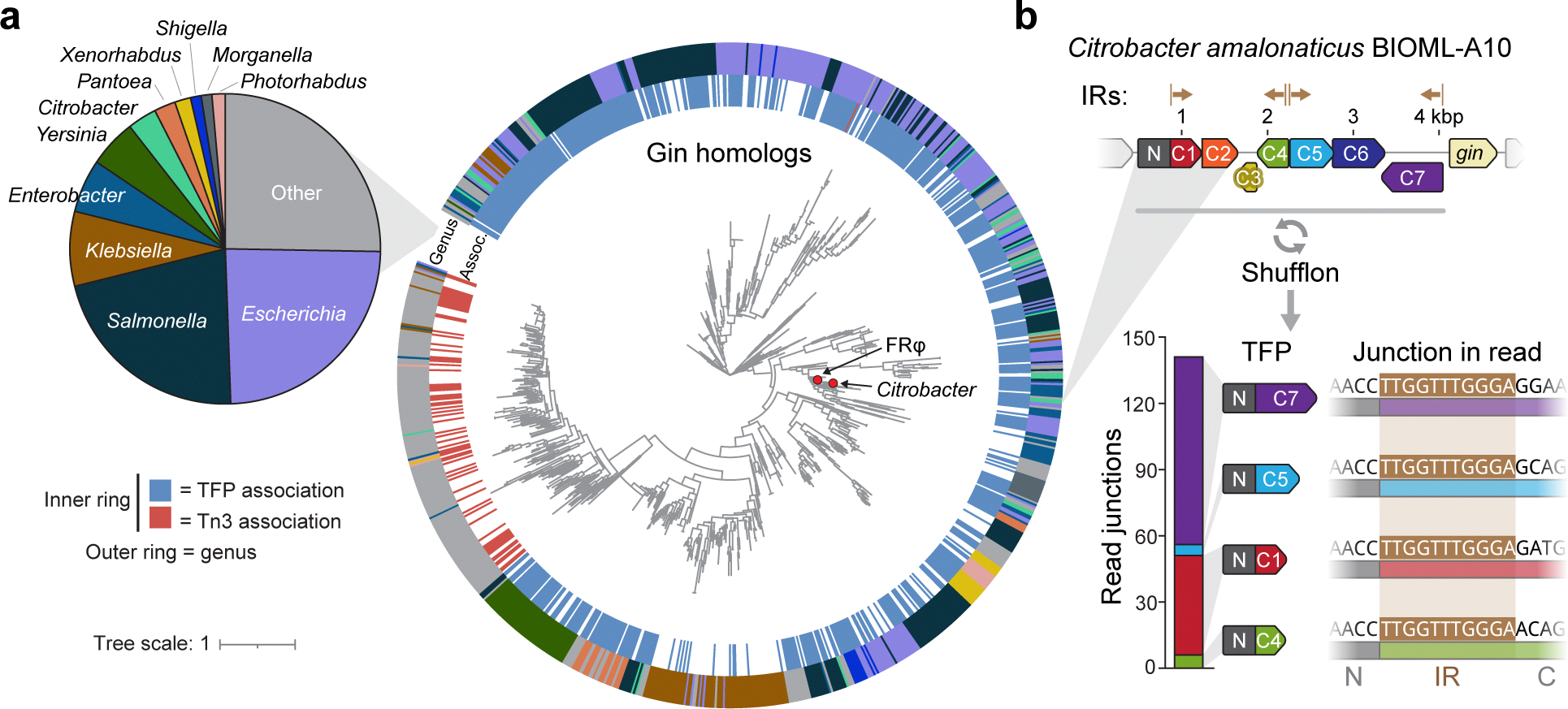


**Figure:**
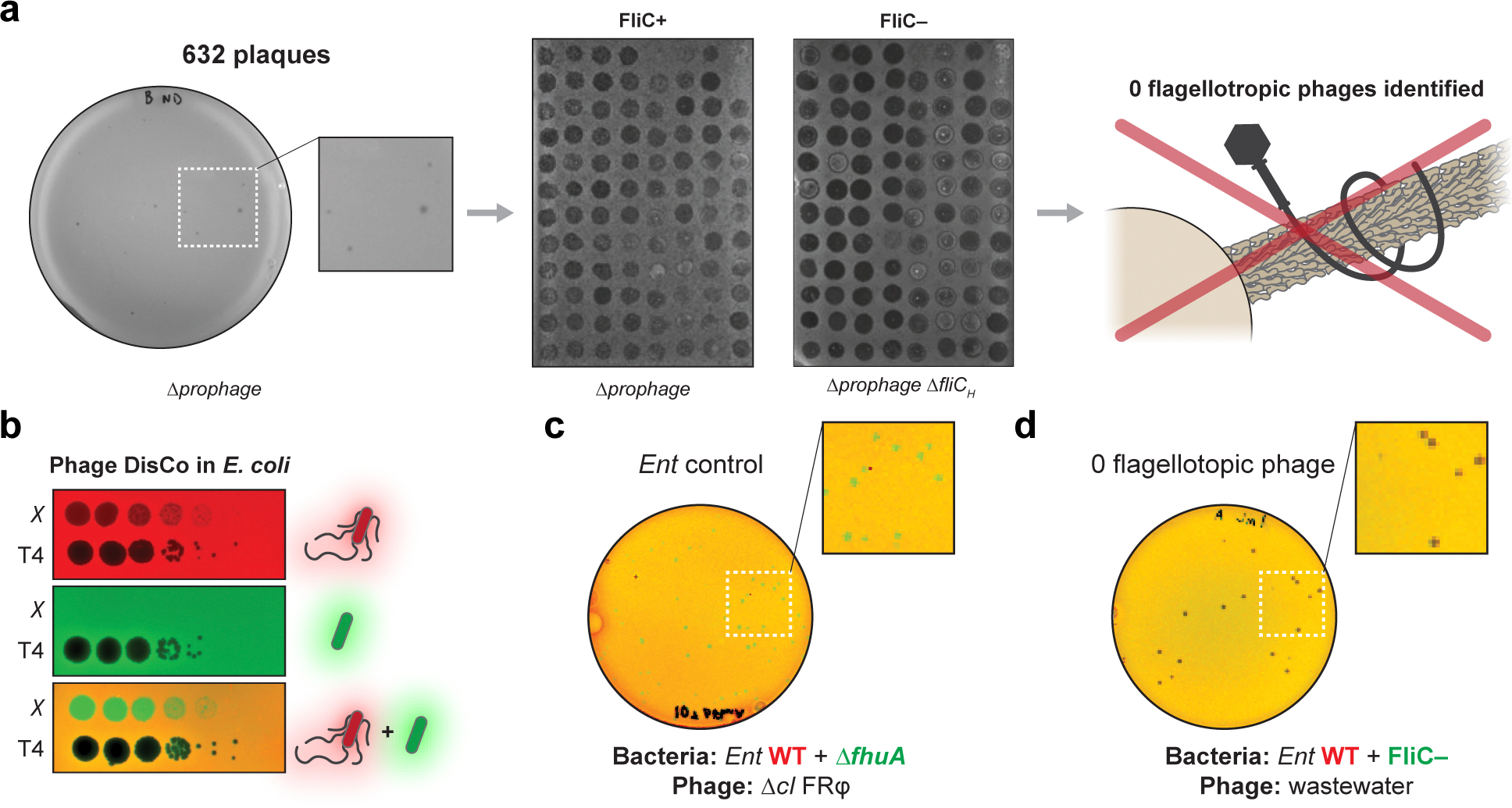


**Figure:**
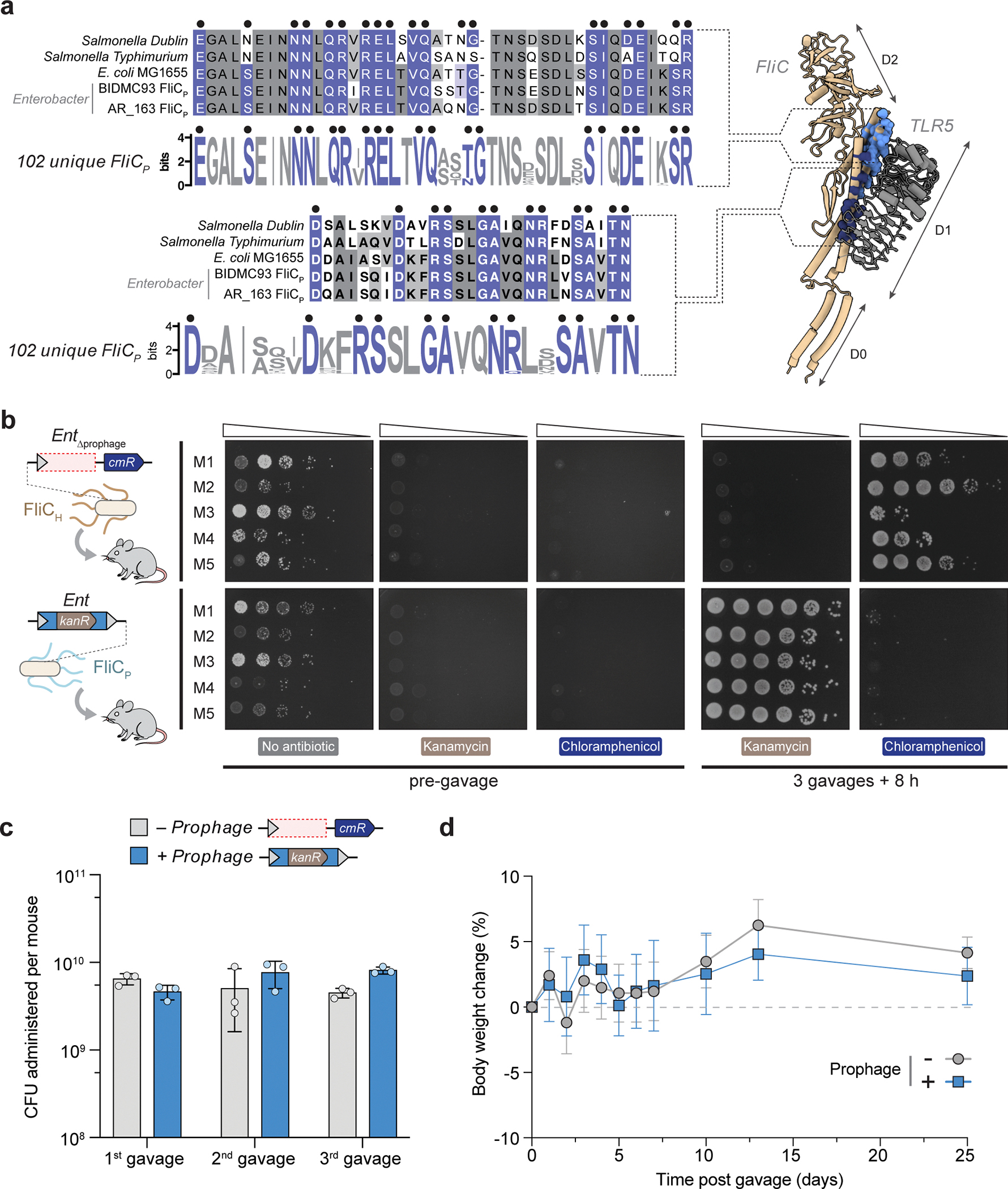


**Figure:**
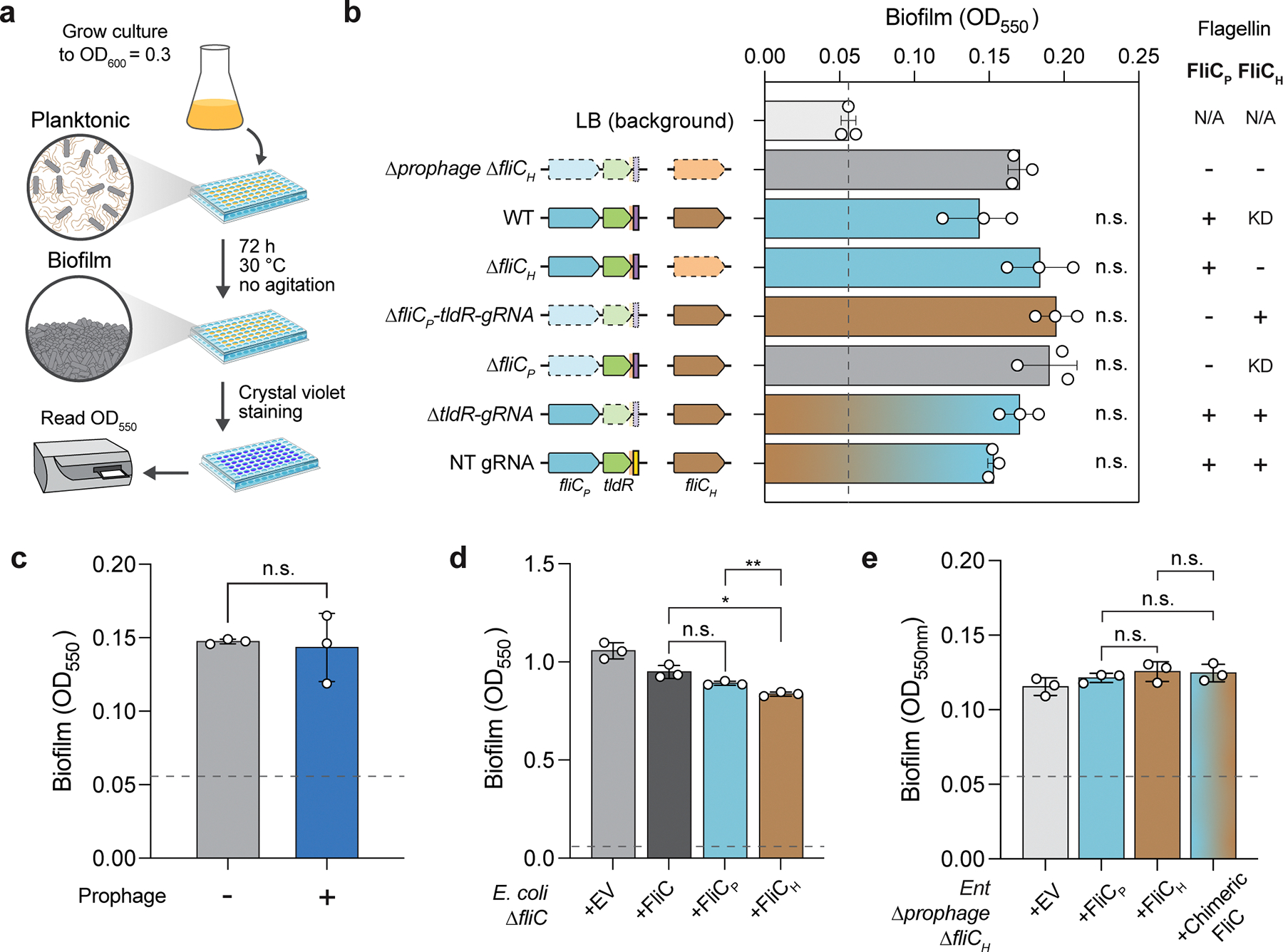


**Figure:**
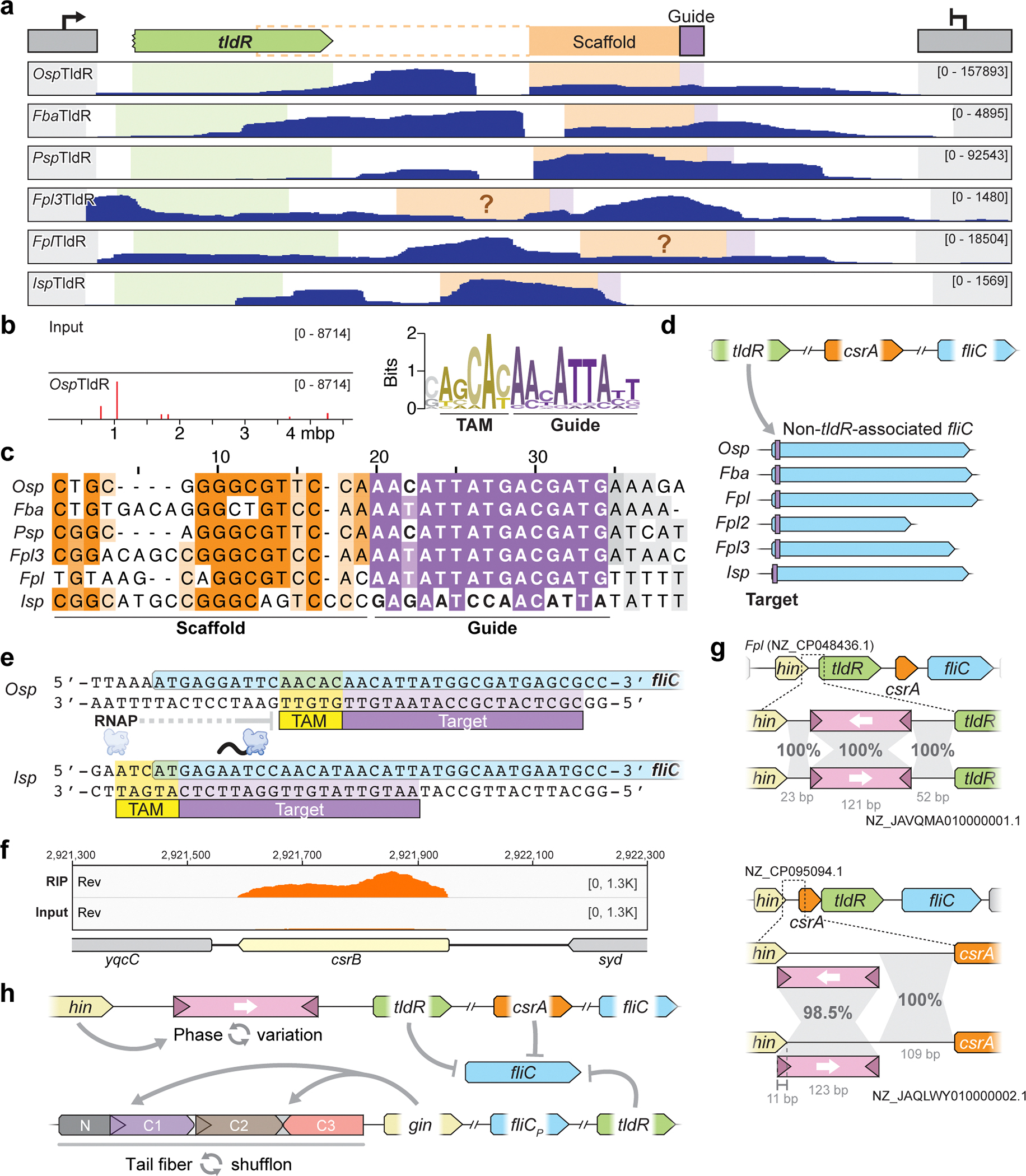


## Supplementary Material

WalkerRichard_etal_SuppTables

## Data Availability

High-throughput sequencing data have been deposited at the National Center for Biotechnology Information (NCBI) Sequence Read Archive (BioProject Accession: PRJNA1256936) and are publicly available.

## CODE AVAILABILITY

All original code has been deposited at Zenodo and is publicly available at DOI 10.5281/zenodo.15307114.
